# Data Preparation for West Nile Virus Agent-Based Modelling: Protocol for Processing Bird Population Estimates and Incorporating ArcMap in AnyLogic

**DOI:** 10.2196/resprot.6213

**Published:** 2017-07-17

**Authors:** Hamid Reza Nasrinpour, Alexander A Reimer, Marcia R Friesen, Robert D McLeod

**Affiliations:** ^1^ University of Manitoba Electrical and Computer Engineering Department Winnipeg, MB Canada

**Keywords:** AnyLogic, shapefiles, ArcMap, West Nile Virus, land cover, bird roosts, bird home range, Manitoba

## Abstract

**Background:**

West Nile Virus (WNV) was first isolated in 1937. Since the 1950s, many outbreaks have occurred in various countries. The first appearance of infected birds in Manitoba, Canada was in 2002.

**Objective:**

This paper describes the data preparation phase of setting up a geographic information system (GIS) simulation environment for WNV Agent-Based Modelling in Manitoba.

**Methods:**

The main technology used in this protocol is based on AnyLogic and ArcGIS software. A diverse variety of topics and techniques regarding the data collection phase are presented, as modelling WNV has many disparate attributes, including landscape and weather impacts on mosquito population dynamics and birds’ roosting locations, population count, and movement patterns.

**Results:**

Different maps were combined to create a grid land cover map of Manitoba, Canada in a shapefile format compatible with AnyLogic, in order to modulate mosquito parameters. A significant amount of data regarding 152 bird species, along with their population estimates and locations in Manitoba, were gathered and assembled. Municipality shapefile maps were converted to built-in AnyLogic GIS regions for better compatibility with census data and initial placement of human agents. Accessing shapefiles and their databases in AnyLogic are also discussed.

**Conclusions:**

AnyLogic simulation software in combination with Esri ArcGIS provides a powerful toolbox for developers and modellers to simulate almost any GIS-based environment or process. This research should be useful to others working on a variety of mosquito-borne diseases (eg, Zika, dengue, and chikungunya) by demonstrating the importance of data relating to Manitoba and/or introducing procedures to compile such data.

## Introduction

**Figure 1:**
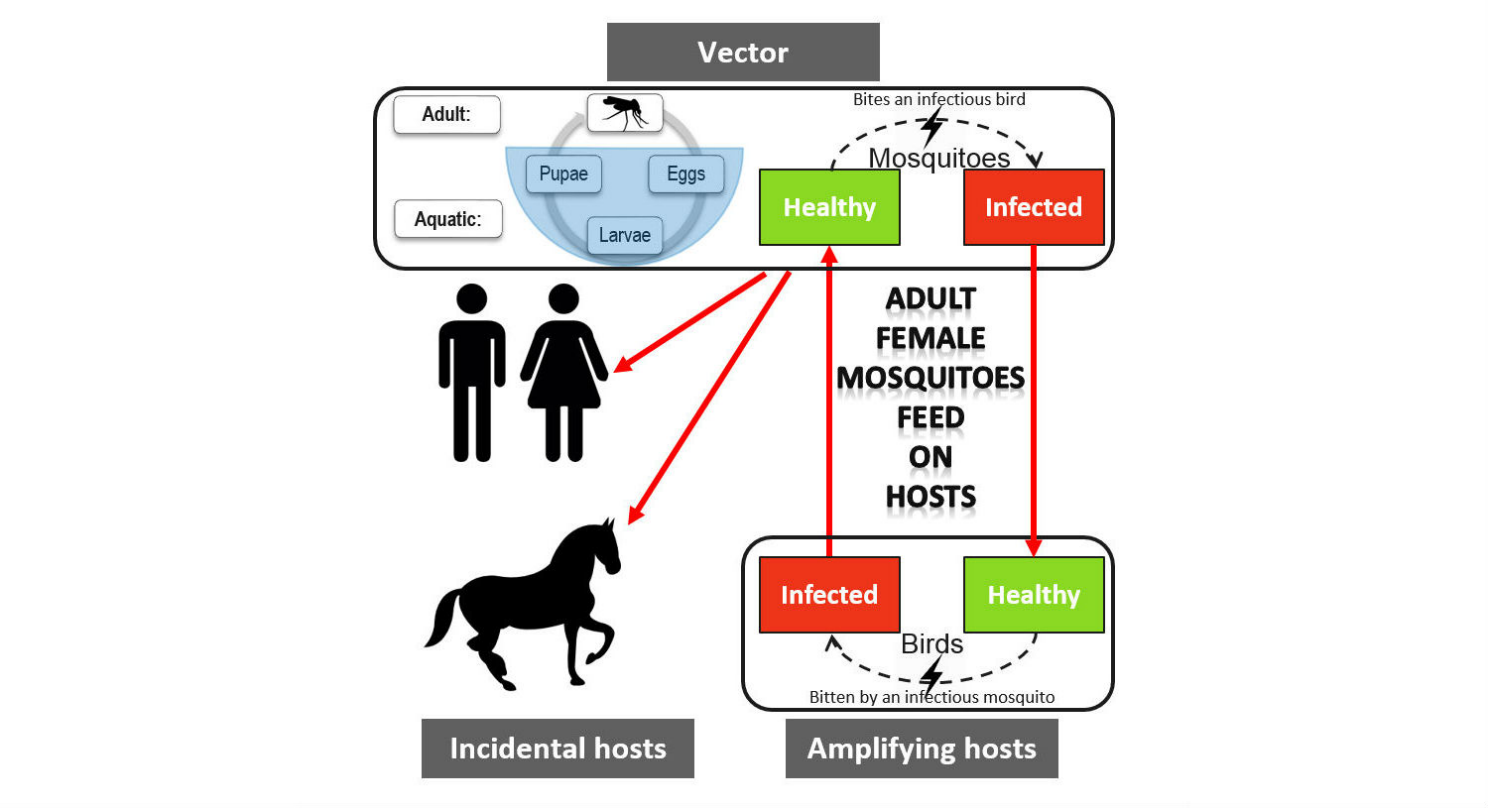
Mosquito life cycle and West Nile Virus transmission cycle diagram.

This paper examines data inputs into an agent-based model (ABM) and simulation of West Nile Virus (WNV) using the AnyLogic software [1], with a specific focus on data collection and compatibility, and preparation or processing techniques. Mosquitoes of certain genera carry and transmit WNV to other animals including humans. Under certain weather and habitat conditions, adult female mosquitoes take a blood meal from their hosts to obtain necessary nutrition to lay their eggs. During the next stages of the mosquito life-cycle, eggs hatch into larvae, and then begin molting their skins until they change into pupae that develop into adult mosquitoes [2]. The main means of transmission and spread of WNV is through birds [3]. An infected mosquito can infect a (healthy) bird by feeding on it. An infected bird can, in turn, infect a (healthy) mosquito that bites the bird. In this transmission cycle, birds act as amplifying hosts since the virus is amplified in their bloodstream, and it could be transmitted to the next group of feeding mosquitoes. Conversely, various kinds of mammals (including humans) act as incidental or dead-end hosts that cannot pass the virus to another host or feeding mosquitoes. The process is shown in Figure 1.

Many scientific studies have examined the transmission dynamic modelling of WNV. Various approaches to model WNV transmission risk or spread are reviewed in Chevalier et al [4]. Most notably, differential equation (DE) models have been utilized to model disease transmission dynamics. Thomas and Urena formulated a difference equation for WNV evolution in a mosquito-bird-human community with a focus on mitigation via pesticide [5]. Wonham et al developed a single-season susceptible-infectious-removed DE model for WNV transmission in a bird-mosquito population [6]. Bowman et al proposed a single-season DE model of WNV transmission dynamics in a mosquito-bird-human population [7]. In this context, ABMs could be deployed to incorporate biodiversity of birds and mosquitoes as completely as possible, along with their interactions with humans. To date, the use of ABMs in the WNV literature has been rather scant, even though it has been extensively employed in many different health care applications [8-13]. In the WNV literature, an ABM was proposed by Li et al [14] for an area of 165 km^2^in Cook County, Illinois, which was modelled as a raster map. Three possible bird species of black-capped chickadee, blue jay, and American crow were considered within this ABM. Another ABM with no human component was proposed by Bouden et al [15] for southern Quebec, Canada. Two bird-biting species of *Culex pipiens* and *Culex restuans*, and two general groups of birds (ie, American crows and the remainder, competent species) were considered within this ABM. In their respective ABM, birds are grouped into roost agents whose moving behavior is modelled with particle systems proposed by Reeves [16], wherein birds’ flight speed and home range are crucial model parameters.

Although AnyLogic is a powerful multi-paradigm modelling framework, there are few user group resources or forums available for its users. To the best of our knowledge, there is only one active user community in LinkedIn for the AnyLogic modelling software. In addition to more traditional simulation, AnyLogic also has relatively recent support for geographic information system (GIS) simulation and modelling. Within GIS, Esri shapefiles are the most commonly used [17-21]. The shapefile format includes vector data representing location, shape, and attributes of geographic features such as lakes, mountains, buildings, and roads.

However, it can be quite difficult to format shapefiles in a way that a modeler could easily apply or use within the AnyLogic framework for GIS-based simulations. This paper explains (in a tutorial-based style) the procedures used to prepare the data required to develop an ABM of WNV spread in Southern Manitoba, Canada. The region of interest is an area of approximately 148,812 km^2^that is partially covered by grasslands (Canadian Prairies), where the primary WNV vector is *Culex tarsalis*.

The value of ABMs is being established through a growing literature base in various fields, but the modelling approach itself is still considered a relative newcomer in relation to mathematical models and more established simulation models of social and human agent systems. ABM is recognized as a computational model that permits a distinctive approach to complementary empirical research [22,23]. In ABMs, dynamic social systems are modelled as a collection of highly stochastic agents (in many cases, primarily humans), their individual profiles or characteristics, their individual behaviors, and interactions between agents and between agents and the environment. Agents are purposeful and autonomous entities able to assess their situation, make decisions, and compete or co-operate with one another on the basis of a set of rules. The conceptual depth of an ABM is derived from its ability to model behavior that may be counterintuitive and/or to discern a complex behavioral whole that is greater than the sum of its parts. ABM provides a natural description of a system that can be calibrated and validated by representative expert agents and is flexible enough to be tuned to high degrees of sensitivity in agent behaviors and interactions. ABMs are particularly well suited to dissipative irreversible system modelling in which agent behavior is complex, nonlinear, stochastic, and may exhibit memory or path-dependence [24,25]. However, the full potential of ABMs remains unrealized as the methods to exploit massive amounts of data are still emerging. The key challenges of an ABM approach applied to socio-ecological systems are improving agent decision and adaption models, improving validation and verification, and improving spatial representation and levels of abstraction [26]. Furthermore, the key challenges for the ABM approach to advance and realize its potential include validating agent behaviors and emergent phenomena, better agent behavioral models, improved simulation analytics, and improving hybrid and large-scale ABMs [27-29].

**Figure 2:**
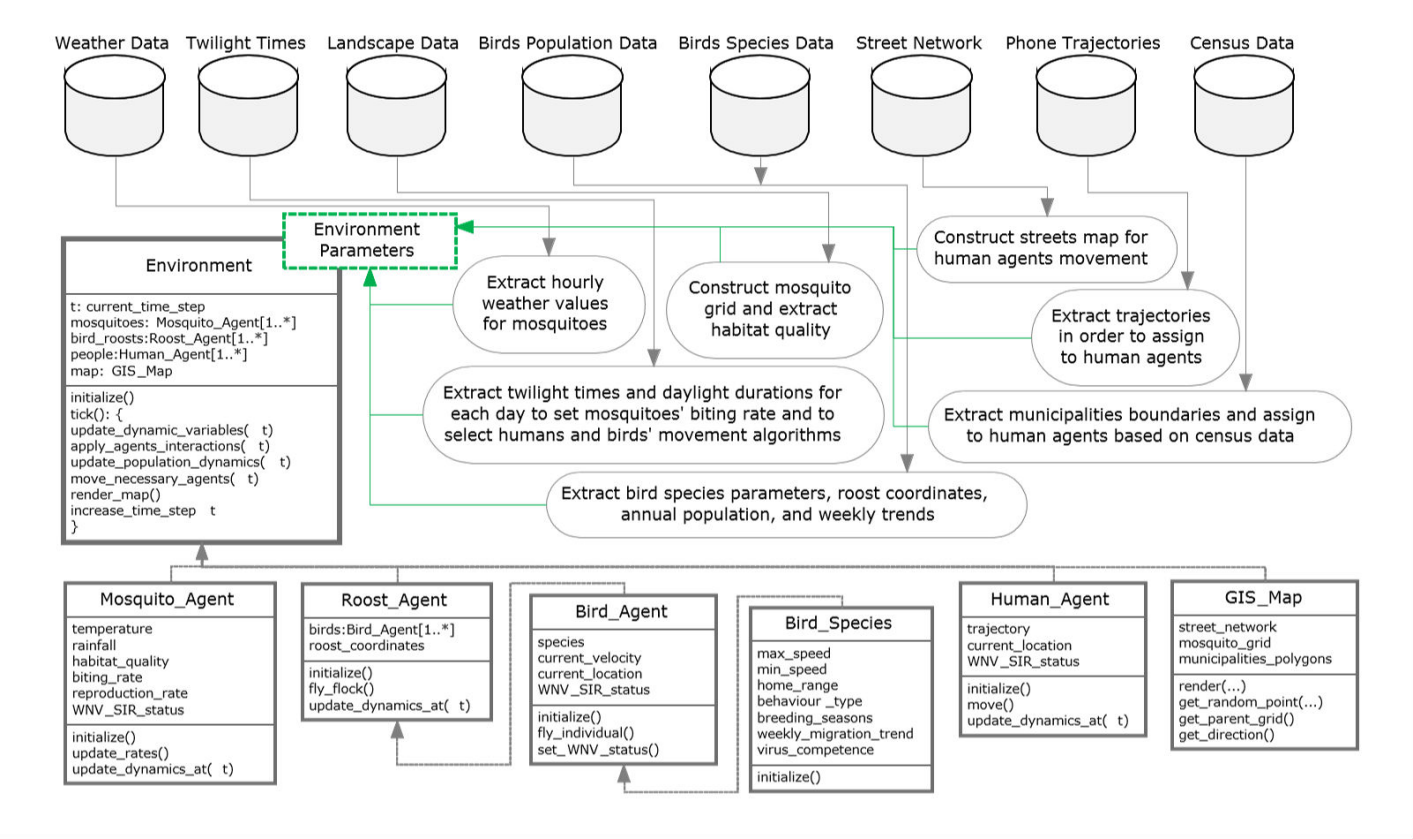
High-level architecture of the ongoing agent-based model.

As noted, WNV is carried and transmitted by mosquito vectors. Birds and humans are among the hosts for the infection. A WNV model requires, at a minimum, data on these three agent types. The mosquito-related data include (but are not limited to) weather for population dynamics, landscape features for habitat preferences, and twilight times and daylight duration for setting peak periods of mosquito agents’ biting activities. A conceptual ABM may model the area as a grid, in which each cell has different properties regarding mosquito population dynamics. Such data would be used to tune or modulate the mosquito parameters of each cell according to weather, landscape, and daylight conditions, ultimately governing mosquito behaviors and interactions. The bird-related data that are collected include nesting/roosting locations, population estimates of each species, home range areas, breeding season months, communal or solitary living habits, and typical flight speeds. A conceptual ABM may distribute and initialize the bird agents of different species based on the population estimates and roosting locations. The movement patterns of birds may be determined based on the home range, flight speed, and living habits of each species. For instance, in each time-step, birds could pick a random flight speed in a certain range and fly up to a certain maximum distance. The algorithms used for movement simulation may be different depending on whether the species are solitary, and whether they are mating at a particular time of the year. The human-related data necessary to incorporate realistic human movement patterns include census counts, street networks, and coordinates of cellular telephone towers providing service for a number of anonymized mobile users, where mobile phones act as proxies for their users. A conceptual ABM may initially distribute human agents over the map based on the census data. Human agents may then move inside the street network according to cellular phone towers or trajectories provided by the data, with cellphones serving as proxies for individuals. The high-level architecture of such an ABM is illustrated in Figure 2. This paper describes and presents the methodology and results for collecting, assembling, and reformatting some of these data for each agent type of a conceptual ABM of WNV propagation.

## Methods

The validity and relevance of any ABM relies on incorporating as much real and meaningful data as possible to characterize the environment and the agents. This section examines the collection and processing techniques of data most relevant for an ABM associated with WNV. This information includes agent data related to mosquitoes, birds, and humans. Similar data processing would be required for other mosquito-transmitted diseases.

The applied techniques are described in some detail, in order to assist others using AnyLogic in combination Esri ArcGIS, which combine to provide a powerful toolbox to modelers, particularly those working on geo-simulations. The details provided here would significantly reduce frustration for other modelers who are beginning to utilize the software, as there is a high level of subtlety in the mechanism/interface of both suites of software. The techniques also illustrate a primary challenge in ABM: specifically, combining different and often disparate datasets.

### Mosquito Data

#### Weather

Preparing weather data is a simple process, and does not need to be discussed in detail; this is primarily due to the familiarity everyone has with weather, as well as readily available data. For this work, one weather station per municipality was considered. The weather data (including precipitation and temperature) for the years 2002-2014 were downloaded from Canada National Climate Data and Information Archive and BioSim databases [30] whenever possible. For the municipalities in which there was no weather station, and for filling in any missing data in the real data, BioSim was used to provide simulated weather data. As an input point for the BioSim built-in simulator [30], whenever possible the coordinates of an existing weather station were used. Otherwise, a location provided by Wikipedia and confirmed on Google maps was used as the coordinates of a municipality. These data were combined with the gathered hourly and daily data. Wherever simulated data were used (in 114 out of 118 municipalities included), the data were flagged for future reference.

#### Landscape

A common approach to describe landscape features is to classify different regions of land into various categories based on ground cover, ground features, and land use. As such, this land feature is called either *land cover* or *land use* classification. These categories or classes may include an urban area, rural area, grassland, agricultural cropland, various forest types, sands, roads, and water. When the classes are defined, remote sensing satellite images (from the Manitoba Remote Sensing Centre in our case) are usually used to classify each region.

This data and other geospatial data are typically available in the shapefile format. The GIS library and components available in AnyLogic have the ability to work with shapefiles. As such, one way to add land-based habitat characteristics for mosquitoes in a WNV model in any GIS-integrated software (such as AnyLogic) is through shapefiles. A square grid shapefile of Southern Manitoba was chosen, where each cell represented a 5 x 5 km^2^mosquito site. Within each cell, the area of each land cover class can be calculated and recorded in the shapefile database file (DBF). The shapefile can then be loaded in AnyLogic. For any given coordinate in the map, one could retrieve the covering mosquito cell and its associated information in the shapefile database. Here, the procedure to create such a shapefile using the Esri ArcMap (part of the ArcGIS software package) is explained in detail. It is noted that the changes in land cover over the span of simulation years are negligible.

First, the land cover data for each region of Manitoba was downloaded from the Manitoba Initiative Data Warehouse [31] in the shapefile format. The class of land cover for each feature (polygon) in the data was identified with a *GridCode* number in the shapefile DBF. The coordinate system of these shapefiles is the Universal Transverse Mercator (UTM), which is a projected coordinate system that enables the ArcMap to calculate geometric properties of a polygon feature such as area or perimeter. For this reason, the coordinate system was kept unchanged at this time.

**Figure 3:**
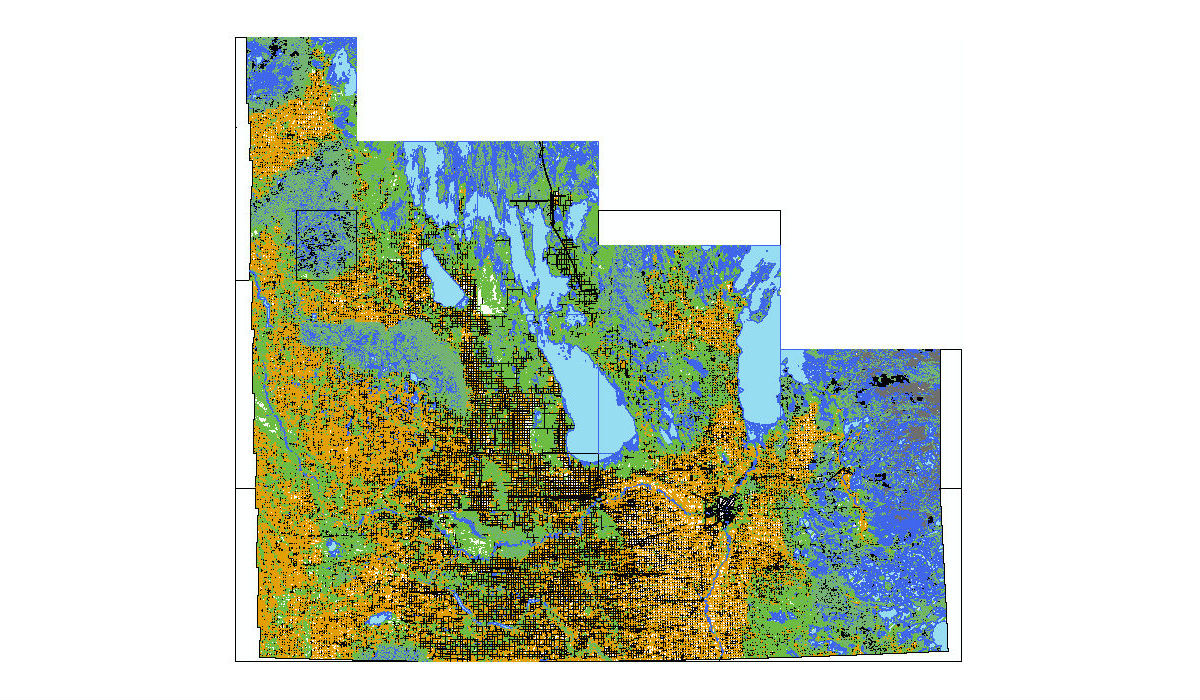
Manitoba Land Cover map (shapefile) generated by combing data from all regions. Blue color represents water body or wetlands; green represents different forest types; orange represents agricultural or forage cropland.

All shapefiles were then added together to make a single general map of land cover using the *append* command in the geo-processing toolbox of ArcMap. Next, the geometry of the new map was repaired using the *features* toolset under the data management toolbox, which is part of the geo-processing toolbox. Repairing the geometry was necessary to fix some common geometry problems (eg, empty parts or duplicate vertices). The outcome was a map (or shapefile) with standard geometric specifications, as shown in Figure 3.

**Figure 4:**
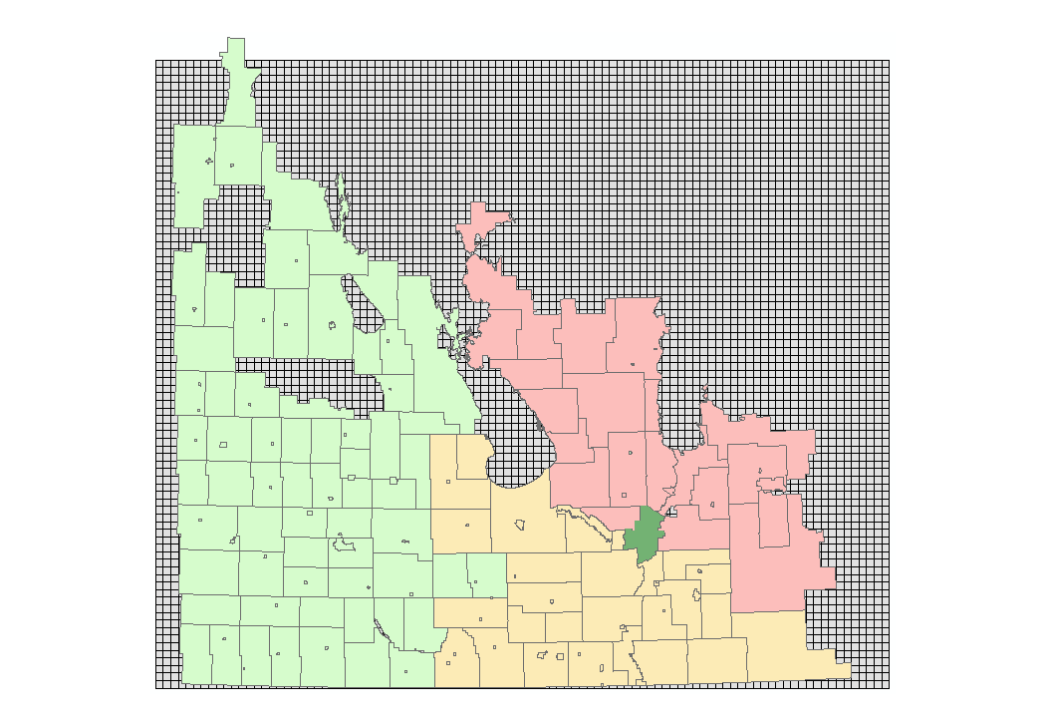
Mosquito grid beneath the map of municipal boundaries. The municipalities are shown (in color) for a better visual clarity of where the mosquito grids are located.

At this stage, the 5 x 5 km^2^mosquito grid had to be built to incorporate the land cover data from the previously prepared shapefile. An overlay grid of southern Manitoba (ie, region of interest) with an accuracy of 5 km x 5 km was created using the *Fishnet* command in the geo-processing toolbox of ArcMap. The output of this procedure was a rectangle-shaped map containing many square cells, as shown in Figure 4. It is noted that the coordinate system of this grid was the UTM, the same as that of the data source and data frame of all the layers in the ArcMap project.

**Figure 5:**
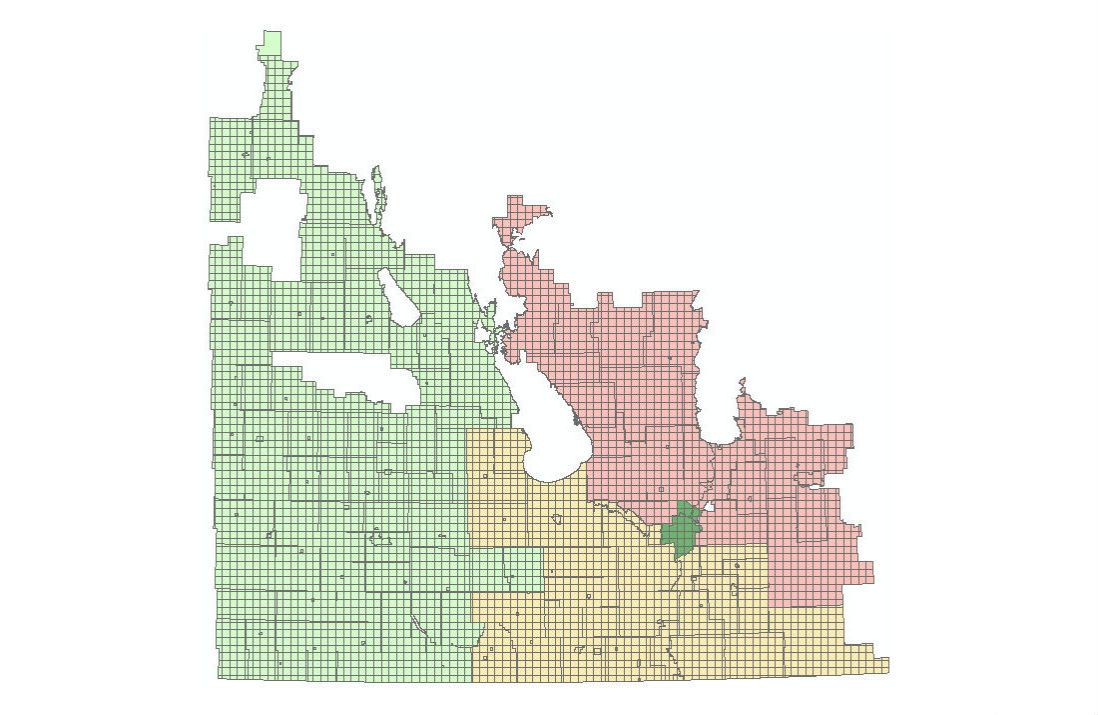
The result of the "identity" command by setting mosquito grid as the "identity feature" and municipal boundaries as the "input feature".

Subsequently, the land cover shapefile from the previous step had to be attached to the mosquito grid. To do so, the *identity* command in the geo-processing toolbox was applied to find the geometric intersection of the grid and land cover map by setting the grid as the *identity feature*, and the land cover map as the *input feature.* As a result, the land cover map (or portions thereof) that overlapped the grid obtained the attributes of the grid, which were basically the square cell identifications (IDs). This means for every single pair of a square cell and a land cover polygon with some overlapping area, a new polygon feature is created in the new map (or shapefile). Figure 5 illustrates an example of this operation. The new map is called the *land cover grid*. For all entries (polygon features) with a known square cell ID in the land cover grid, the land cover *GridCode* is also known. For the next step, the geometric area of each of these entries is required. Therefore, a new field called *Area_Sq_M* was added to the shapefile database using the *attribute table* in the ArcMap. The area of each feature in square meters was then calculated and stored in this field using the *calculate geometry* feature in the *attribute table*.

There are ways to find the proportional area of each land cover class for each square cell using ArcMap. Once these metadata are calculated, they can be stored in the shapefile DBF format, and can be accessed in a classical FoxPro database query. However, there are two techniques that a developer should consider. First, the filename plus its extension must be less than eight characters. Second, there is only one table per DBF, so the table name in a Structured Query Language (SQL) *select* command is the same as the filename, and the database address in the connection string only includes the location without the filename. A sample Java code is provided in Multimedia Appendix 1, illustrating how to connect to a shapefile DBF using a Miscrosoft (MS) Access dBase driver connection string.

Given the limitation of shapefile databases, a decision was made to clone the shapefile database into an MS access database, and apply necessary queries and adjustments there. As such, the land grid shapefile database was exported into a text file, and the text file was imported into an MS Access dBase. Using a small C# application, the proportional area of each land cover class within each square cell was then calculated and stored in an MS Access table where the primary key was the square cell ID. This means that for any given mosquito cell, if the cell ID is known, a simple SQL query could reveal the exact information regarding the land cover within the cell. The AnyLogic GIS library is helpful in these instances. Once a shapefile is loaded into a GIS map component of AnyLogic, for any given pair of latitude and longitude, the ID (or any other attribute) of the shapefile feature at the same point is accessible using the *findPoliticalArea* function. In our case, AnyLogic was set to return the square cell ID for the polygon feature over a given coordinate. The cell ID can, in turn, be used to query land cover information of the area.

The last step is to prepare the mosquito grid shapefile to be loaded into AnyLogic. For this, the mosquito grid must be clipped to reduce the number of unnecessary cells in which no information about the land cover is present. First, a mask of all the cells with useful information is created to clip out the remaining cells. The mask creation procedure is as follows: (1) the *dissolve* command from the geo-processing tool is applied by setting the land cover grid (including the grid and land cover data) as the input, and (2) the “Create multipart feature” option is unchecked; no field is added to the *dissolve* or *statistics* fields.

**Figure 6:**
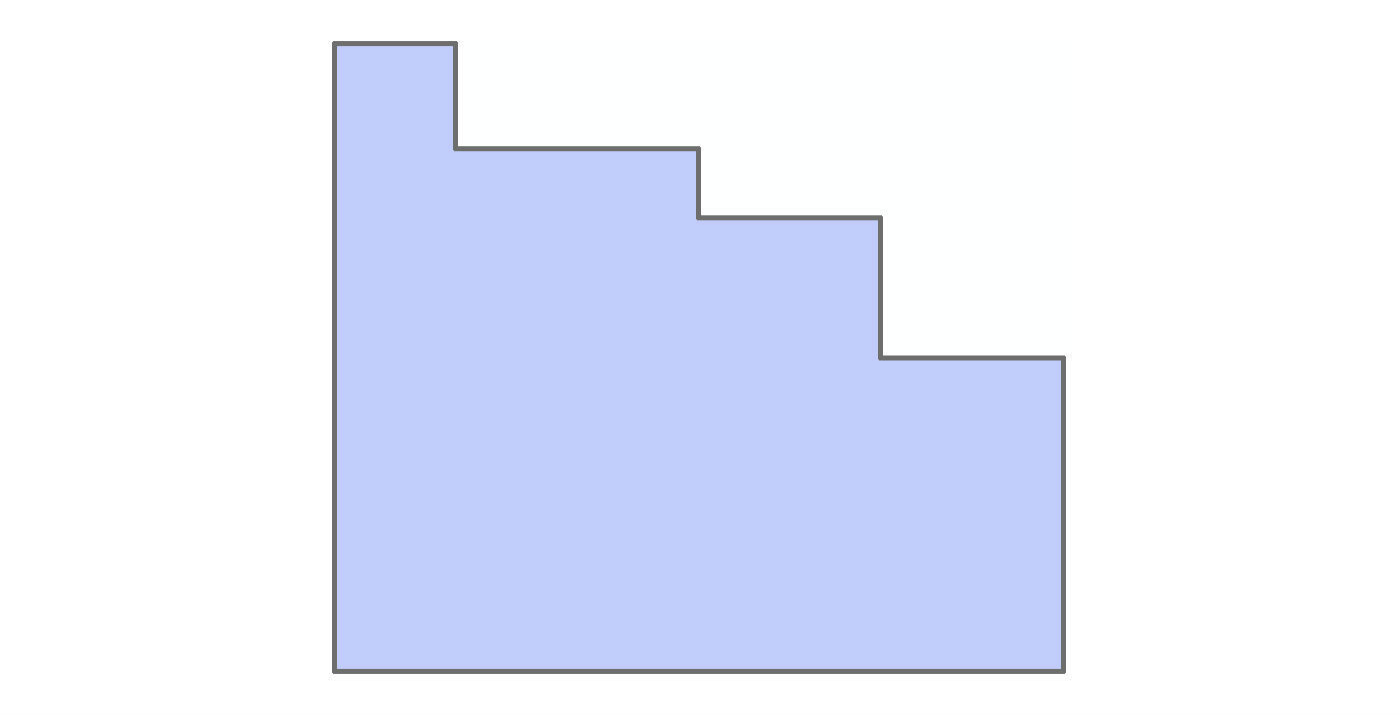
The boundary mask of land cover grid. The area indicates locations where the land cover data are known.

This would give a single polygon for the whole map of the land cover grid, which could be used as the boundary of the region of interest. At this stage, a hole was noticed in the single boundary (mask) polygon which was due to missing data in the land cover map. As such, a filling donut holes procedure was necessary: (1) the *editor toolbar* was added to the ArcMap toolbar, then an edit session was started by selecting the *editor toolbar*; (2) the mask in the *create features* bar was selected as the active layer, then a template as the *construction tool* from the box below it was selected; (3) a rectangle or a polygon over the donut hole was drawn, then edits were saved and the edit session ended; and (4) once again the shapefile was dissolved to merge all polygons in this shapefile together. At this stage, the mask without any holes was ready, as shown in Figure 6.

To exclude the unnecessary mosquito cells, either the *clip* or *intersection* command must be applied on the mask and the mosquito grid. It is noted that while the *intersect* method saves a copy of feature ID (FID) fields of both shapefiles in the new shapefile, the *clip* method keeps no record of FIDs. Therefore, if one uses the *clip* method, the FID of each cell of the grid (ie, mosquito cell ID) should be copied into a new field beforehand.

Finally, the coordinate system must be projected from UTM to World Geodetic System 1984 so that it is consistent with AnyLogic. One more vital technique is that the shapefile must have at least two fields (other than the default FID and Shape) so that it can be loaded within AnyLogic, in particular for use with the *findPoliticalArea* function.

### Bird Data

#### Population Estimate

Many different data sources had to be combined to produce the bird population database. Since detailed population maps were not readily available for most species, a process had to be developed to estimate the population of individual species within relatively small areas. Two approaches were developed to create these estimates. The first was a “top-down” approach, which relied heavily on population estimates for large regions and relative abundance maps for local distributions. The second method could be considered a “bottom-up” approach. This approach used localized point count surveys and species-specific correction factors to estimate population. Using these two separate approaches, it was possible to establish population estimates that were suitable for our models. As with many ABM approaches attempting to use as realistic and meaningful data as possible, best guess estimations were required. As more accurate data becomes available, the veracity of the estimates improves.

#### Partners In Flight Approach

**Figure 7:**
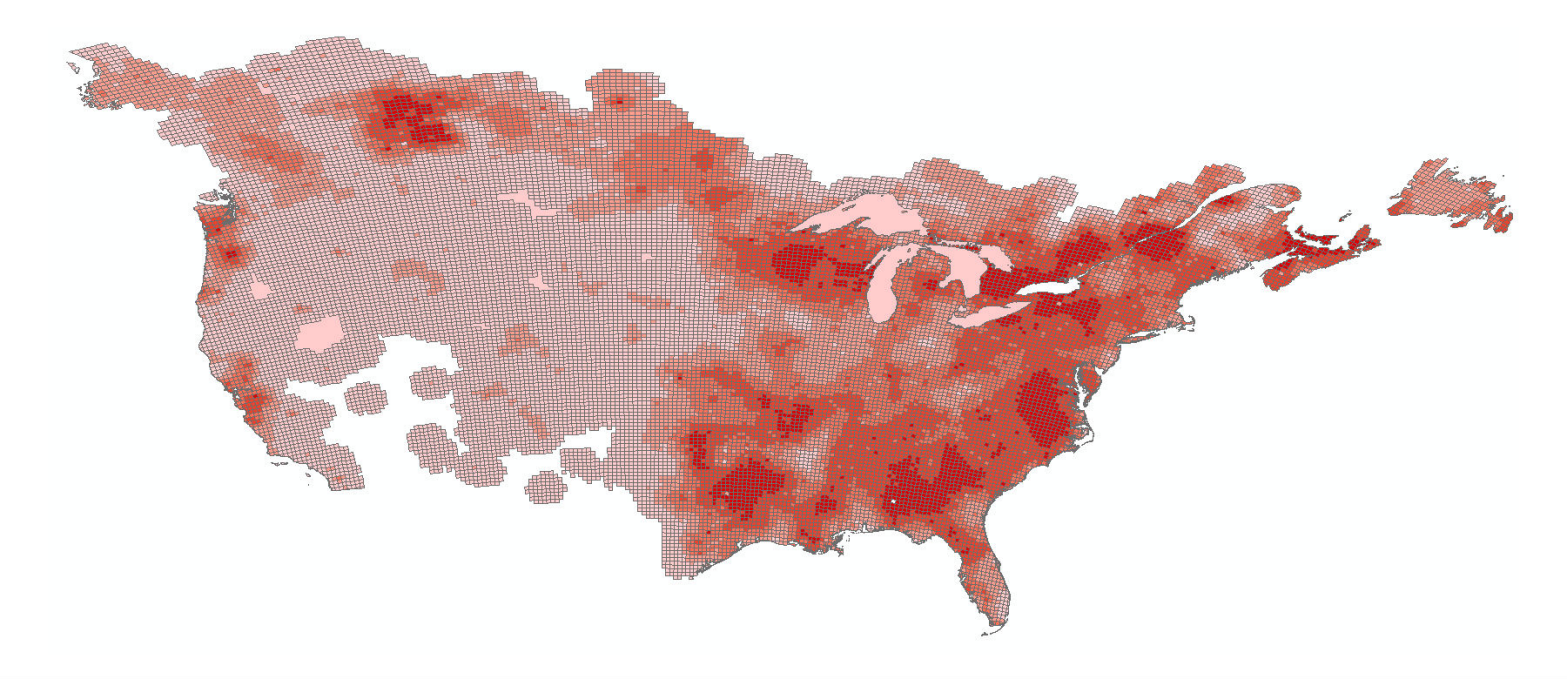
An example of the USGS abundance map. The darker areas denote areas of high species abundance.

The first “top-down” approach used the United States Geological Survey (USGS) abundance maps, which were created by USGS from their 50 roadside stops breeding bird surveys that were conducted at peak breeding season (June for most species) [32]. The files were downloaded from the website in the form of shapefiles [32]. Each cell in these maps contained a relative abundance value representing the average number of birds observed by the survey in that area. The data from these surveys had been extrapolated and processed so that a map of the entire United States and Southern Canada was available (see Figure 7).

These abundance maps were then combined with a 10 x 10 km^2^grid, provided by the Manitoba Breeding Bird Atlas (MBBA) [33]. This was done to make the data compatible with the breeding location data from the MBBA. The grid was downloaded as a Keyhole Markup Language Zipped file from the MBBA website, and combined with the abundance map shapefiles using a Python script in the Esri ArcMap.

**Figure 8:**
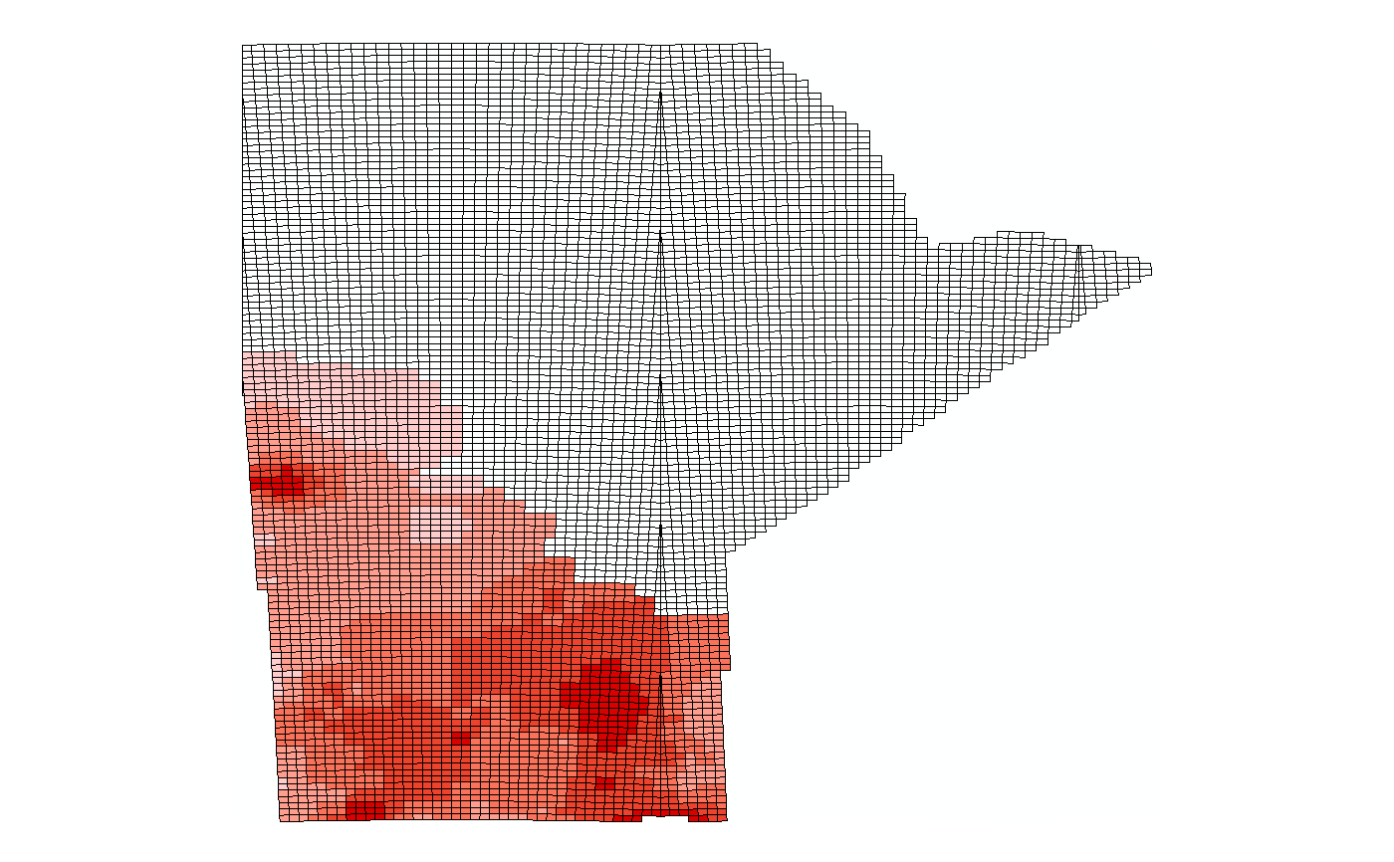
United States Geological Survey abundance data for Manitoba quantized into a 10 km by 10 km grid.

Each 10 km x 10 km square received the relative abundance value of the abundance map cell that covered it. If a square was covered by parts of two or more cells, the relative abundance value was taken as the weighted mean between all cells values, with more weight being given to those cells that covered the majority of the 10 km x 10 km square. If the square was not completely covered by the abundance map cells, the parts that were not covered were considered to be covered by a cell with a relative abundance value of zero. This value assumed that the species in question did not live beyond the edges of the abundance map. In many places, this will have been a valid assumption, but at the edges of the USGS study area, this may have caused an underestimation of abundance. In this regard, the relative abundance data was combined with the MBBA 10 km x 10 km squares as shown in Figure 8.

**Figure 9:**
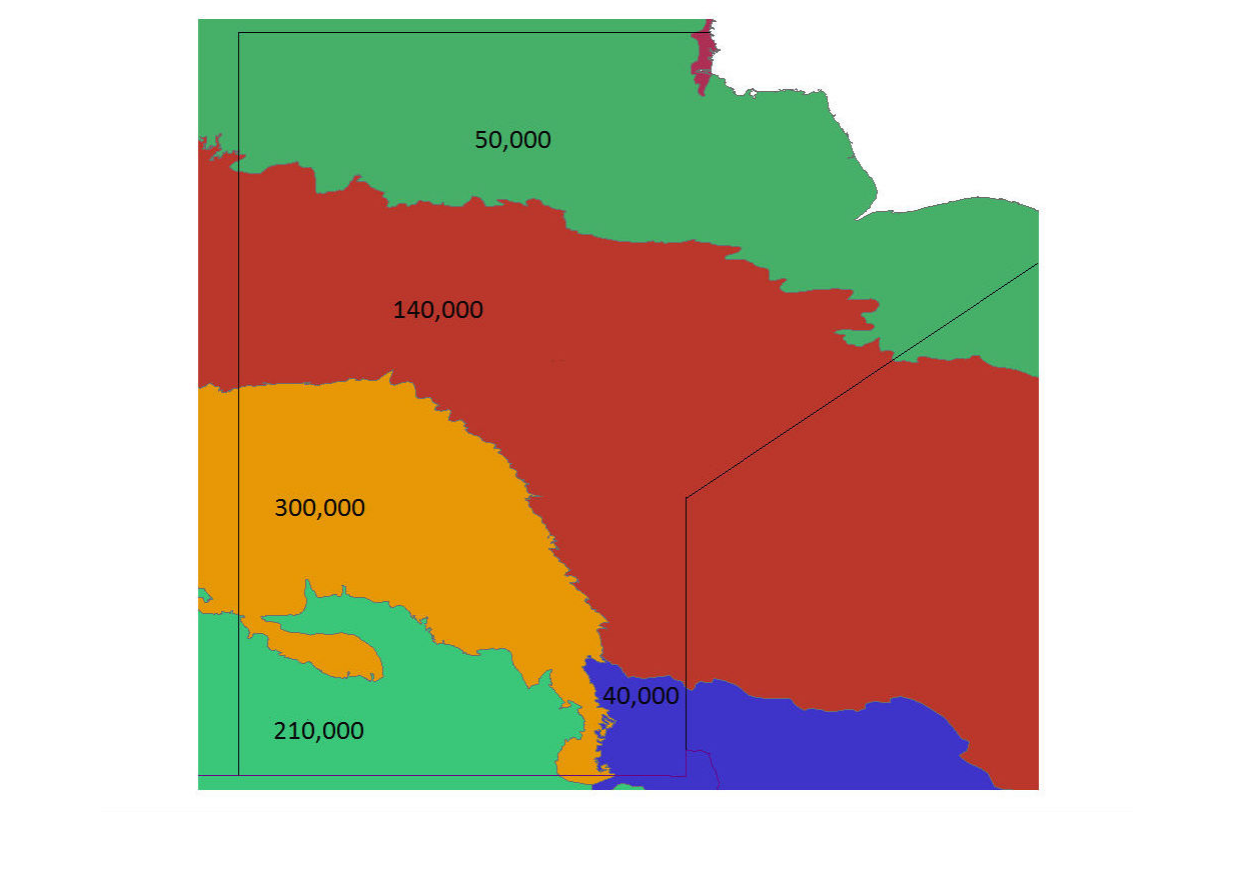
Bird Conservation Regions in Manitoba, with example population estimates for each region. Population estimates did not include birds in the same Bird Conservation Region outside of Manitoba.

Next, the relative abundances were combined with the Partners in Flight (PIF) population estimates for regions in Manitoba [34]. Each PIF population estimate was an estimated population of a certain species for an individual Bird Conservation Region (BCR) in Manitoba. The study area contained three BCR regions, and the PIF gave population estimates for each of the three regions for all species (see Figure 9 [35]). The population was distributed between the squares in each region to create real population estimates for each square. A greater population was given to squares with a higher relative abundance. Some squares were not exactly 10 km x 10 km, so a greater population was also given to the larger squares. The equation for the estimated real population of each square is provided in Figure 15.

To achieve an idea of how the population changed over the course of a year (mostly due to migration), the population estimates of each square were combined with weekly abundance estimates of bird species in Manitoba, made available by the Manitoba Naturalist Society [36]. The population estimate was assumed to be the population at the time of maximum abundance in June when the USGS point counts were conducted. From this value, the rest of the data were scaled accordingly. In this way, the single population estimate was extrapolated over the year.

Finally, the squares were filtered by whether or not the bird species bred in that area. Using the breeding status data provided by the MBBA, it was possible to remove each square that did not contain a nesting or roosting area within it. Considering the requirements of the WNV ABM, no differentiation was made between roosting and breeding areas.

An assumption was made that by filtering out squares after dividing population estimates, the number of birds in each square was underestimated. However, efforts to divide the population after filtering by breeding status led to negligibly different results (<0.5%), since most squares where a species was found contained breeding, so this effect could be ignored.

#### Boreal Avian Modelling Approach

The population was also estimated using a second process, the “bottom-up” approach. Using this approach, we began with point count data that was provided by the MBBA. This data was only for squares where breeding was suspected, and gave a larger sample size than the USGS abundance maps had within Manitoba. The MBBA point counts were conducted by a participant standing in several predetermined locations inside each 10 km x 10 km square who recorded the number of birds that they observed or heard within 5 minutes. This system did not record all birds in the area, but it did give a relative index into how many birds were in the area. To convert the point count data into a real population estimate for the square, correction factors needed to be applied.

Correction factors were obtained from the Boreal Avian Modelling (BAM) project [37]. Although BAM takes many different and complex correction factors into consideration as they create their own population estimates, only two are considered here: the effective detection radius (EDR) and singing rate. The EDR is defined as the distance from the point of observation where as many birds were detected beyond this radius as were undetected within this radius [37]. This factor took into consideration the fact that a bird further away would be less likely to be detected, and that certain species would be harder to detect at further distances. Thus, when the point count data were considered, it was reasonable to assume that the point count numbers correlated to the number of birds within the EDR.

The singing rate was given as the rate at which a bird sang out per minute, or similarly the proportion of a bird population that sang at least once in one minute. Since most point counts depend on hearing the sound of a bird more than seeing it to identify the species, the singing rate gives a useful approximation of how many birds remain quiet and thus undetected during the point count. By multiplying the singing rate by the number of minutes spent in observation, one can find the proportion of birds that sang out and had a chance of being identified in the point count. If the point count was long enough, this proportion would rise above 100% as birds began to sing more than once. However, the observer for each point count was trained to count individual birds, not individual bird songs, so multiple bird songs by the same bird could be discarded [38]. Therefore, the singing rate-time product was capped at 100%. The population estimate for each square was calculated as shown in Figure 16.

After calculating population estimates for each square, the population was again modulated to show annual changes. This was done in the same fashion as the first “top-down” approach using the annual abundance data. In this way, both approaches were used to create usable population estimates for the model.

#### Species Data

Other data that were collected and inferred from a diverse variety of sources regarding each species are reported in Multimedia Appendix 1. These data include home range area, breeding season months, communal or solitary living habits, and typical flight speeds.

The spring/summer home range of all birds was considered to be a circular area. As such, the average radius in meters was calculated and reported in Multimedia Appendix 1. The home range of a given species depends heavily on the habitat and food abundance conditions. The top priority was to collect the data from Manitoba. If possible, reported mean home ranges for the landscapes neighboring the province were considered. When there were no data for similar landscapes, the mean of the home ranges (as reported in different sources and weighted by the sample sizes) was used. It was also desirable to avoid underestimating the average home range, as the ongoing ABM required the ceiling of an average home range to be set as the maximum flight distance for birds.

Breeding timing and living habit data were primarily collected from the Birds of North America online database [39]. The breeding season range goes up to, but does not include, the end month. Living habits were categorized into three groups: solitary roosting behavior, year-round communal roosting, and semiannual communal roosting. Semiannual communal roosting included species that roost individually or in pairs during the breeding season, and then form flocks for migration in the fall. In general, the roosting behaviors of birds are often difficult to categorize. These designations represent an estimate on which type of model each species’ behavior may best fit in the specific ABM, and are not intended to reflect a universal definition of communal roosting.

Mean flight speed is reported in the format of meters per second in Multimedia Appendix 1. For many species, the data on flight speed were either very sparse or nonexistent. For such species, certain approximations had to be made, such as using the reported speed for a similar species of that genus or taking the average of reported speeds of the whole family. It was decided to find the typical flight speed at which birds fly/forage during a day; however, in most sources it was unclear what type of speed was measured. Values were generally either *minimum power speed* (*Vmp*) or *maximum range speed* (*Vmr*). If a certain species had both *Vmp* and *Vmr* available, the smaller *Vmp* would have been recorded as the flight speed of the species. Whenever there were only reports on the maximum flight speed, the minimum number in the given range of maximum flight speeds was used as the typical speed. It is notable that a bird’s normal flight speed going from perch to perch is much less than the numbers reported in Multimedia Appendix 1. These values were treated as the ceiling of typical speed in the birds’ movement component of the ongoing ABM, and the birds’ minimum flight speed was set at two meters per second.

### Human Data

**Figure 10:**
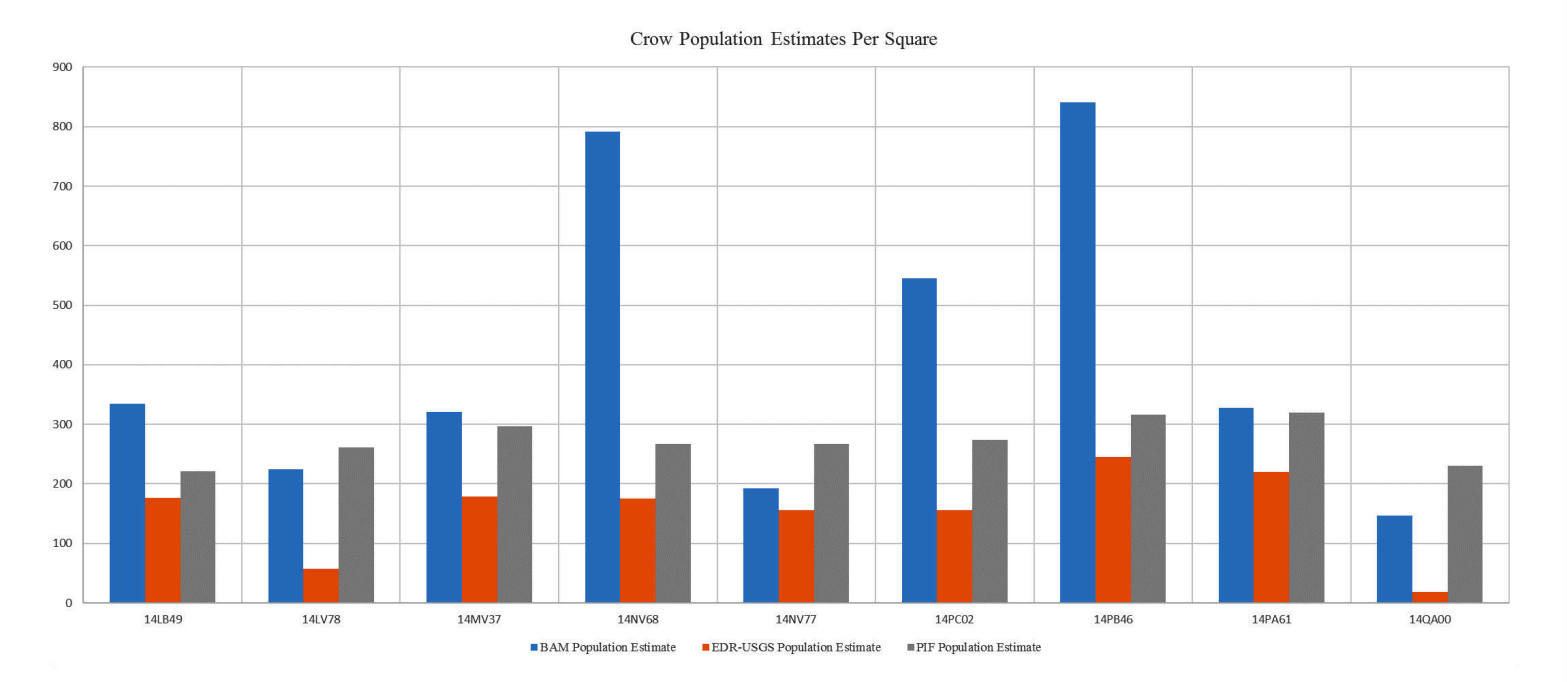
Comparison of crow population estimates between the Boreal Avian Modelling (BAM) and Partners In Flight (PIF) methods in some selected squares. EDR-USGS: Effective Detection Radius-United States Geological Survey.

The Open Street Map of the province was downloaded from GeoFabrik.de as a highly compressed Protocol-Buffer Binary Format file. The street map was then extracted from this binary file using the routing features of AnyLogic. A sample street map of Southern Manitoba including only trunk, primary, and secondary roads can be seen in Figure 10. The A* pathfinding algorithm was applied on this network for human agents’ routing. Census data for each municipality of the province for initial location of human agents were downloaded from Statistics Canada. Municipal boundaries in Manitoba were downloaded as a shapefile from the National Resources Canada website. The population of human agents then had to be distributed within these boundaries according to the census data for each municipality. AnyLogic has a *GISRegion* component, in which one can call the function *randomPointInside* to randomly choose a point inside the given region. Therefore, this function can be used to initialize the human agents’ population inside each municipality. However, the shapefile polygon features, representing municipal boundaries, first had to be converted to AnyLogic *GISRegion(s)*. Up to AnyLogic version 7.2, the built-in converter was not fully functional to convert all the shapefile features to AnyLogic *GISRegion(s)*. A sample AnyLogic Java code for this nonintuitive conversion can be found in Multimedia Appendix 1. The code also gives hints to developers on how to extract all point coordinates of objects in a shapefile. It is notable that each shapefile has a different number of nested layers of objects. This may be the reason why the built-in converter does not work for all shapefiles.

For the purpose of this paper and brevity, human movement patterns, data collection, and processing methods are not discussed further, but these factors are integral to ongoing WNV agent-based modelling. In addition to the readily available census data mentioned above, other sources include those related to personal cellular devices, as well as technologies being developed for intelligent transportation systems. Earlier proofs of concept were demonstrated in prior work [13,40,41]. A further extension of this paper will discuss the preparation and manipulation of these data sources.

## Results

### Mosquito Data

The final weather database had daily and hourly values of weather temperature and rainfall for 118 rural and urban municipalities in Manitoba from 2002 to 2014. This dataset could be used for any weather-dependent studies in the Southern Manitoba region. The final land cover database had information on 6067 square cells in Southern Manitoba. For each cell, the corresponding municipality and weather station, total area, coordinates, and area of different land cover classes present in the cell were known. The land cover classes used in this dataset include Agricultural Field, Deciduous Forest, Water Body, Range and Grassland, Mixed-Wood Forest, Wetland-Marsh, Wetland-Treed Bog, Treed Rock, Coniferous Forest, Fire-Burnt Area, Open Deciduous Forest/Shrub, Agricultural-Forage Crop, Cultural Features, Forest Cut Block, Sand and Gravel, Roads and Rail Lines, Wetland-Fen, and Lichen health. The land cover database can be used in geo-spatial studies in Southern Manitoba. Such studies are of particularly high importance for agricultural purposes. One limitation of the land cover database and the extraction procedure is that the land cover was assumed to be static. Generally, more work on land cover (and in particular on dynamic land cover changes) is ongoing. For instance, Murray-Rust et al proposed an open-frame ABM to capture changes in land cover [42]. As future work, the land cover extraction procedure could be automated using Python scripts in ArcGIS. Such an automated procedure makes the system adjustable in response to changes in land cover data. The land cover database, in conjunction with the weather database, could benefit studies that focus on the forecasting of mosquito-borne diseases in Sothern Manitoba.

Future work on weather data may focus on improving algorithms used for simulating missing data, particularly hourly rainfall values for a specific area, if hourly estimates are necessary. In this study, the BioSim software was applied for this purpose, which produced estimates of hourly rainfall values. Depending on the fidelity of the application, one may need to include more weather stations in Manitoba by going through the same procedures explained earlier.

### Bird Data

The final bird database contained information on 152 different bird species. For each species, there were population estimates for each of the 2056 square cells, which were roughly 10 km x 10 km areas located in Southern Manitoba. Each of these population estimates was also used to create a weekly population estimate for each square to represent weekly impacts of migration. Only squares in which some evidence of breeding had been found were included for each species, as these squares were assumed to also contain nocturnal roosts for the species [15]. This dataset could also be used by other researchers working on topics such as modelling birds’ movements, bird interactions in various ABMs, and geo-simulations including birds.

**Figure 11:**
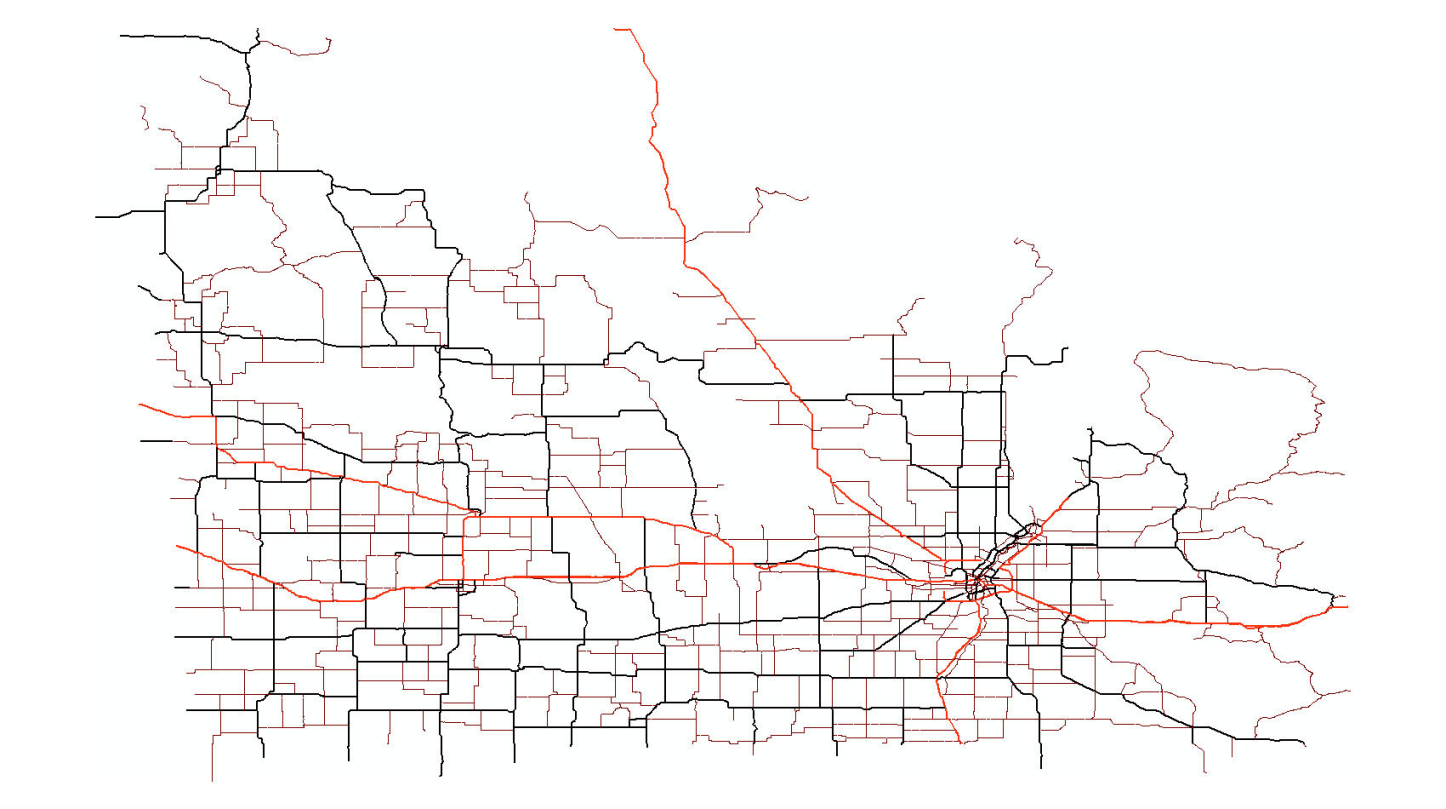
Southern Manitoba street network showing only trunk, primary, and secondary roads.

**Figure 12:**
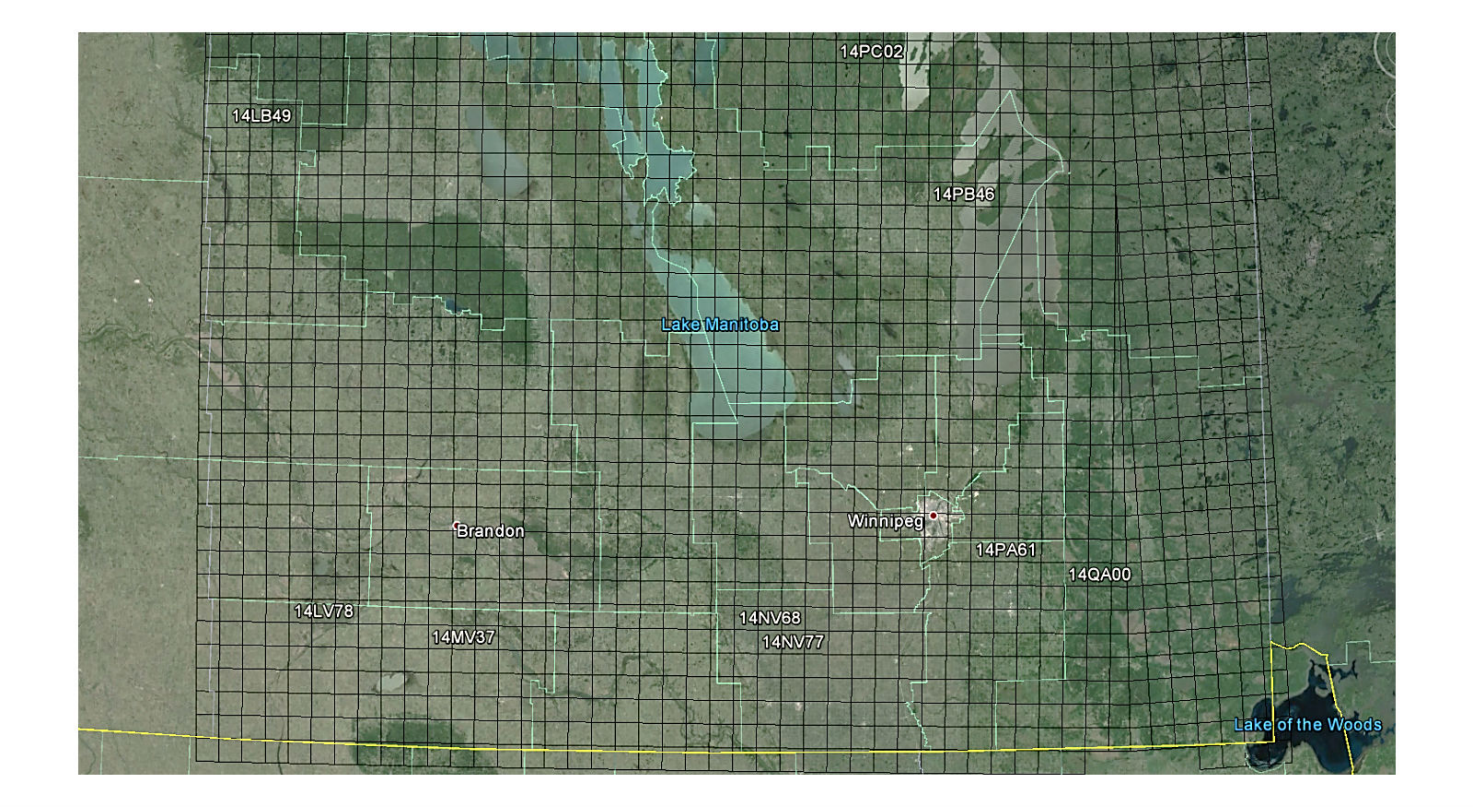
Physical location of some selected squares of bird roosts.

**Figure 13:**
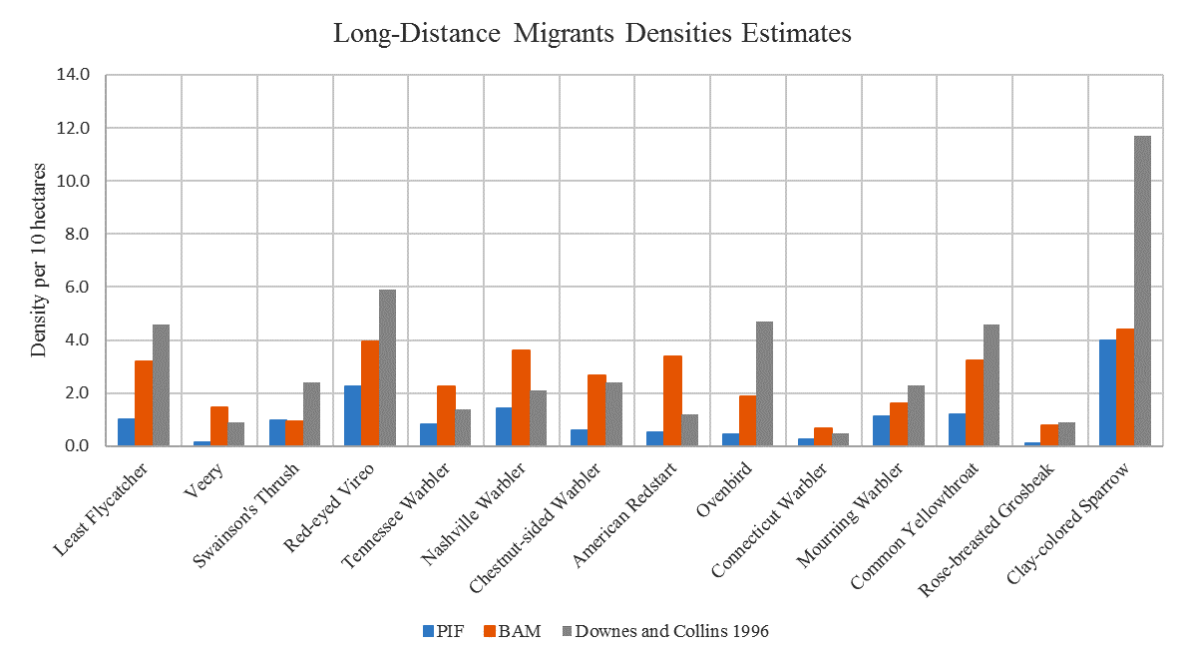
Comparison of the mean densities of Boreal Avian Modelling (BAM) and Partners In Flight (PIF) against densities in Downes and Collins for a number of long-distance migrant species.

**Figure 14:**
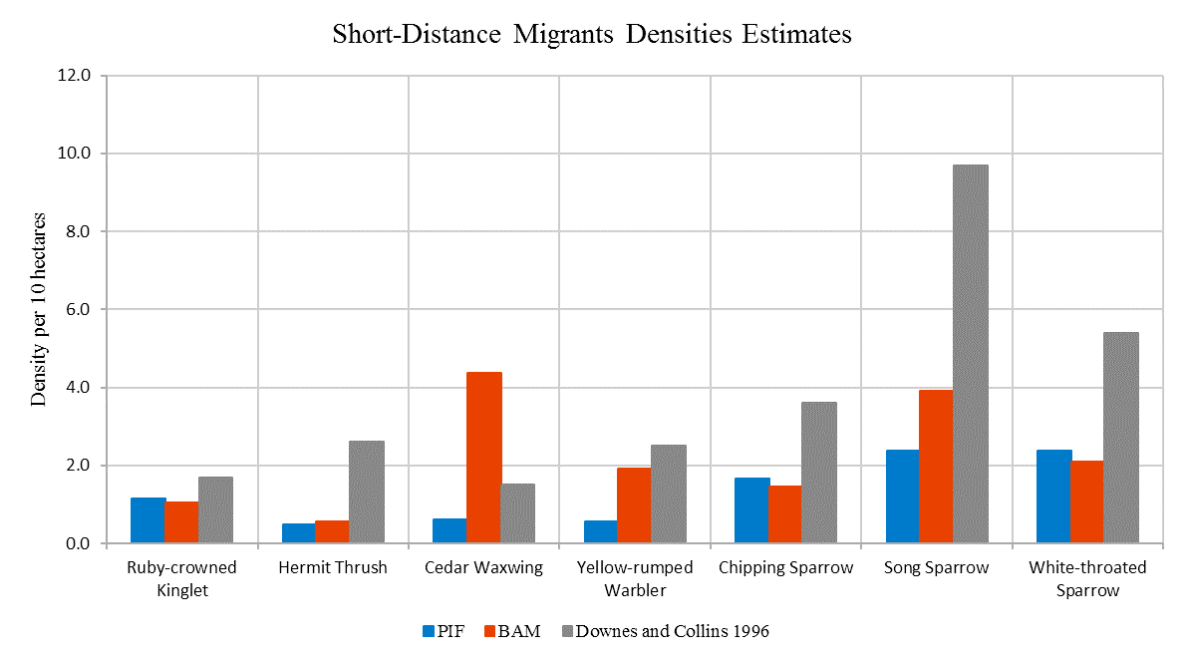
Comparison of the mean densities of Boreal Avian Modelling (BAM) and Partners In Flight (PIF) against densities in Downes and Collins for a number of short-distance migrant species.

Due to fundamental differences between the two population estimation approaches, their assumptions, and availability of data, the two estimates show a degree of disparity in some areas. The chart in Figure 11 shows a comparison of population estimates of American crow species for various locations (squares) in Manitoba. The BAM population estimates were calculated using the methodology previously discussed. The EDR-USGS estimates in Figure 11 were calculated in a similar manner, but used the USGS 50 stops data for point counts instead of the MBBA point counts. The PIF population estimates, as mentioned before, were calculated from the regional PIF estimates and abundance maps created from the USGS 50 stops data. The similarity between the EDR-USGS and PIF estimates in most of the squares reveals the importance of the availability of stops and point counts data. Notably, the difference between the two estimates in the other squares confirms the differences between the “top-down” and “bottom-up” approaches. Figure 12 shows the physical location of selected squares in Figure 11. To achieve an idea of how the two estimates compare against some historical data in Manitoba, an average density for a number of species was calculated from the average BAM and PIF estimates for the species across the province. In Figure 13 and Figure 14, these densities are compared against the Manitoban bird densities calculated for 1966-1994 by Downes and Collins [43] and reported in the work of Kirk et al [44] for two groups of species of short-distance and long-distance migrants. If a species winters mostly within Canada or North America, it is categorized as a short-distance migrant. If the birds mainly spend winters in Central or South America, they are considered long-distance migrants [44]. In general, for the long-distance migrants, the BAM densities have less disparity from the birds’ densities provided by Downes and Collins [43], compared to the PIF. Conversely, for the short-distance migrants, the PIF densities are closer to the birds’ densities provided by Downes and Collins [43].

Both bird population estimate approaches have several assumptions and shortcomings built in. The “top-down” approach used USGS abundance maps that were based on a very small sample size (<2% of the birds’ project area, or approximately 4112 of 205,600 km^2^), and these points had only been surveyed approximately once per year (50 stops per year). Additionally, the PIF regional population estimates, although useful for large-scale conservation efforts and approximations [34], are not made to be accurate at a small scale. Thus, the first approach contains rather sparse data that has been heavily processed and extrapolated to pertain to a large detailed area. The “bottom-up” approach sought to fix some of these problems, and was based on the MBBA point counts, which were available for all of the squares over several years. These squares were again not surveyed regularly over the span of a year (15 times over 5 years) but much more of the study area was covered by these point counts [38]. The correction factors used to convert point counts into population estimates were also (in part) designed by BAM to make up for the weaknesses in the PIF population estimates. However, the correction factors used here did not take every factor into account that could have influenced the point counts. The EDR (and possibly the singing rate) would have varied with different habitats and vegetation types, but the approach described here did not take this into account. The EDR has a large effect on the population estimate, and is consistently smaller than the radius used in the PIF estimates. As a result, the population estimates are likely to be higher than the PIF estimates. There is also likely a habitat bias against certain hard-to-reach habitats. The point counts were done by volunteers, and mostly beside roads, so the more remote locations were less likely to be surveyed. This factor may have caused an over- or under-representation, depending on the species and its preferred habitat. In addition to this problem, there is evidence that point counts done next to roads obtained biased results for some species. More work on correction factors, and population estimates in general, is ongoing and future studies and data should be able to improve on these processes.

Bird species data, including home range area, breeding season months, communal or solitary living habits, and typical flight speeds, are presented in Multimedia Appendix 1. As mentioned in the Methods section, as well as Multimedia Appendix 1, many of the reported species data are estimations of some kind. Therefore, many more field studies and work on bird species (although improving) are consistently required.

**Figure 15:**
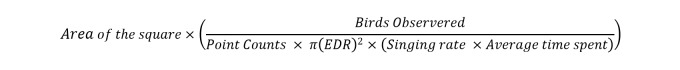
Equation to estimate real population of bird species using the Boreal Avian Modelling (BAM) approach.

**Figure 16:**
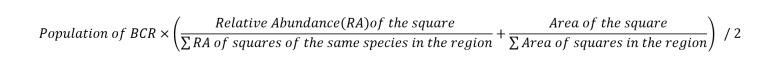
Equation to estimate real population of bird species using the Partners In Flight (PIF) approach.

### Human Data

The procedures explained in this paper (for preparing the human data) have two outputs. The first output details a database of human census in municipalities of Manitoba with their boundary coordinates. These coordinates are saved as AnyLogic *GISRegion* objects on file. The second output involves the street network of the province in a compatible format with AnyLogic GIS components. A future extension of this paper will discuss issues regarding the extraction of trajectories for human agents according to cellular phone tower data.

## Discussion

### Conclusions

AnyLogic simulation software, in combination with Esri ArcGIS, provides a powerful toolbox for developers and modelers to simulate almost any GIS-based environment or process. In this paper, the application of interest was WNV propagation in the province of Manitoba. The land cover data of Manitoba was rasterized in an optimum sized shapefile compatible with AnyLogic. Some hints and techniques regarding working with shapefiles in AnyLogic were reviewed. A database of over 150 different bird species vulnerable to WNV, including their nesting locations, population estimates, home range radii, roosting behavior, and start and end of the breeding season, was collected. The street network for Manitoba, extracted from OpenStreetMap, was loaded into AnyLogic to be used in its pathfinding library. The procedures for collecting, combining, and reformatting all these data are explained in detail in a tutorial-based style to benefit other modelers working in similar areas.

Researchers are constantly exploiting new nontraditional sources of data for modelling different human diseases. For example, in a relatively recent study, Google search data have been used for modelling transmission dynamics of the Zika virus [45]. On the contrary, in this paper, more traditional data sources were gathered and prepared in a suitable way for agent-based modelling of WNV. However, the final proposed dataset could also be used to model mosquito population dynamics (eg, to evaluate control strategies). Some recent studies in this area can be found in the work of Ewing and Cobbold [46] and Marini et al [47]. Inevitably, modelling natural or environmental processes depends heavily on the availability of appropriate data; this is particularly true for verification and validation of models, as models and simulations would not gain significant attention unless they are shown to closely resemble reality. This resemblance can only occur with meaningful data; therefore, the importance of data cannot be overstated. The pertinent procedures and an overview of resultant data for WNV geo-modelling are presented in this paper.

### Limitations

There are some limitations in our data mining and assembling procedures, each of which was discussed in the corresponding section. Notwithstanding, similar mining methods could be adopted by other researchers to compile such datasets according to their own specific needs for other geographic areas (eg, estimating population and location of birds in other provinces/states in North America). This research should be useful to others working on a variety of mosquito-borne diseases (eg, Zika, dengue, and chikungunya) by providing the data relating to Manitoba and/or a systematic path to follow for producing and processing such crucial data. Unlike WNV, these viruses generally only survive in humans. WNV has been able to permanently establish itself in the United States because it can survive in humans, horses, and birds, giving the virus a wider variety of hosts. However, the requirement for modelling human movement patterns, weather, and habitat is still equally important, as any WNV model is tightly correlated with these components.

## Appendix

Multimedia Appendix 1 Sample codes and birds species data.

## Abbreviations

ABM: Agent-Based Model
BAM: Boreal Avian Modelling
BCR: Bird Conservation Region
DBF: DataBase File
DE: differential equation
EDR: Effective Detection Radius
FID: Feature ID
GIS: Geographic Information System
ID: identification
MS: Microsoft
MBBA: Manitoba Breeding Bird Atlas
PIF: Partners In Flight
SQL: Structured Query Language
USGS: United States Geological Survey
UTM: Universal Transverse Mercator
Vmp: minimum power speed
Vmr: maximum range speed
WNV: West Nile Virus

## References

[1] Borshchev A (2013). The Big Book of Simulation Modeling: Multimethod Modeling with AnyLogic 6.

[2] (2016). American Mosquito Control Association.

[3] Reed K, Meece J, Henkel J, Shukla S (2003). Birds, migration and emerging zoonoses: west nile virus, lyme disease, influenza A and enteropathogens. Clin Med Res.

[4] Chevalier V, Tran A, Durand B (2014). Predictive modeling of West Nile virus transmission risk in the Mediterranean Basin: how far from landing?. Int J Environ Res Public Health.

[5] Thomas D, Urena B (2001). A model describing the evolution of West Nile-like encephalitis in New York City. Math Comput Model.

[6] Wonham MJ, de-Camino-Beck T, Lewis MA (2004). An epidemiological model for West Nile virus: invasion analysis and control applications. Proc Biol Sci.

[7] Bowman C, Gumel AB, van den Driessche P, Wu J, Zhu H (2005). A mathematical model for assessing control strategies against West Nile virus. Bull Math Biol.

[8] Silverman BG, Hanrahan N, Bharathy G, Gordon K, Johnson D (2015). A systems approach to healthcare: agent-based modeling, community mental health, and population well-being. Artif Intell Med.

[9] Bilge U, Saka O (2006). Agent based simulations in healthcare. Stud Health Technol Inform.

[10] Friesen MR, McLeod RD (2014). A survey of agent-based modeling of hospital environments. IEEE Access.

[11] Thompson D, Cullen KW, Redondo MJ, Anderson B (2016). Use of relational agents to improve family communication in type 1 diabetes: methods. JMIR Res Protoc.

[12] Neighbour R, Oppenheimer L, Mukhi SN, Friesen MR, McLeod RD (2010). Agent based modeling of “crowdinforming” as a means of load balancing at emergency departments. Online J Public Health Inform.

[13] Laskowski M, Demianyk BC, Witt J, Mukhi SN, Friesen MR, McLeod RD (2011). Agent-based modeling of the spread of influenza-like illness in an emergency department: a simulation study. IEEE Trans Inf Technol Biomed.

[14] Li Z, Hayse J, Hlohowskyj I, Smith K, Smith R (2005). Agent-based model for simulation of West Nile virus transmission. http://mysite.science.uottawa.ca/rsmith43/AgentbasedmodelWNV.pdf.

[15] Bouden M, Moulin B, Gosselin P (2008). The geosimulation of West Nile virus propagation: a multi-agent and climate sensitive tool for risk management in public health. Int J Health Geogr.

[16] Reeves W (1983). Particle systems---a technique for modeling a class of fuzzy objects. ACM SIGGRAPH Comput Graph.

[17] Dahal K, Chow T (2014). A GIS toolset for automated partitioning of urban lands. Environ Model Softw.

[18] Fecht D, Beale L, Briggs D (2014). A GIS-based urban simulation model for environmental health analysis. Environ Model Softw.

[19] Formetta G, Antonello A, Franceschi S, David O, Rigon R (2014). Hydrological modelling with components: A GIS-based open-source framework. Environ Model Softw.

[20] Laranjo L, Rodrigues D, Pereira AM, Ribeiro RT, Boavida JM (2016). Use of electronic health records and geographic information systems in public health surveillance of type 2 diabetes: a feasibility study. JMIR Public Health Surveill.

[21] Frew PM, Archibald M, Schamel J, Saint-Victor D, Fox E, Smith-Bankhead N, Diallo DD, Holstad MM, Del RC (2015). An integrated service delivery model to identify persons living with HIV and to provide linkage to HIV treatment and care in prioritized neighborhoods: a geotargeted, program outcome study. JMIR Public Health Surveill.

[22] Epstein J (2012). Generative Social Science: Studies in Agent-Based Computational Modeling.

[23] Axelrod R, Tesfatsion L (2006). Appendix A: a guide for newcomers to agent-based modeling in the social sciences. Handbook of Computational Economics.

[24] Helbing D (2012). Agent-based simulations and experiments to study emergent social behavior. Social Self-Organization.

[25] Bonabeau E (2002). Agent-based modeling: methods and techniques for simulating human systems. Proc Natl Acad Sci U S A.

[26] Filatova T, Verburg P, Parker D, Stannard C (2013). Spatial agent-based models for socio-ecological systems: Challenges and prospects. Environ Model Softw.

[27] Bankes SC (2002). Agent-based modeling: a revolution?. Proc Natl Acad Sci U S A.

[28] Hahn H (2013). The Conundrum of Verification and Validation of Social Science-based Models. Procedia Comput Sci.

[29] Macal C (2016). Everything you need to know about agent-based modelling and simulation. J Simulation.

[30] Régnière J, St-Amant R, Béchard A (2014). Natural Resources Canada.

[31] (2017). Government of Manitoba.

[32] Sauer J, Hines J, Fallon K, Pardieck D, Ziolkowski J, Link WA (2015). The North American Breeding Bird Survey, Results and Analysis 1966 - 2013 Version 01.30.2015.

[33] (2015). Bird Studies Canada.

[34] Rich T, Beardmore C, Berlanga H, Blancher P, Bradstreet M, Butcher G (2013). Partners in Flight North American Landbird Conservation Plan.

[35] (2014). Bird Studies Canada on behalf of the North American Bird Conservation Initiative.

[36] Carey B, Christianson W, Courcelles A, Cuthbert C, de March L, Smet KD (2006). Finding Birds In Southern Manitoba.

[37] (2010). Boreal Avian Model Project.

[38] (2010). Manitoba Breeding Bird Atlas.

[39] (2015). The Birds of North America Online.

[40] Neighbour R, Mukhi SN, Friesen MR, McLeod RD, Crowley M (2012). Vehicular Traffic Modeling Governed by Cellular Phone Trajectories. http://ieeexplore.ieee.org/document/6398876/.

[41] Borkowski M, McLeod RD (2008). Epidemic Modeling with Discrete Space Scheduled Walkers. http://ieeexplore.ieee.org/document/4616694/.

[42] Murray-Rust D, Robinson D, Guillem E, Karali E, Rounsevell M (2014). An open framework for agent based modelling of agricultural land use change. Environ Model Softw.

[43] Downes C, Collins B (1996). The Canadian Breeding Bird Survey, 1966-94. Canadian Wildlife Service Progress Note No. 210.

[44] Kirk DA, Diamond AW, Smith AR, Holland GE, Chytyk P (1997). Population Changes in Boreal Forest Birds in Saskatchewan and Manitoba. Wilson Bull.

[45] Majumder MS, Santillana M, Mekaru SR, McGinnis DP, Khan K, Brownstein JS (2016). Utilizing nontraditional data sources for rear real-time estimation of transmission dynamics during the 2015-2016 Colombian Zika virus disease outbreak. JMIR Public Health Surveill.

[46] Ewing DA, Cobbold CA, Purse BV, Nunn MA, White SM (2016). Modelling the effect of temperature on the seasonal population dynamics of temperate mosquitoes. J Theor Biol.

[47] Marini G, Poletti P, Giacobini M, Pugliese A, Merler S, Rosà Roberto (2016). The role of climatic and density dependent factors in shaping mosquito population dynamics: the case of Culex pipiens in northwestern Italy. PLoS One.

