# Supplementary material for: Data Preparation for West Nile Virus Agent-Based Modelling: Protocol for Processing Bird Population Estimates and Incorporating ArcMap in AnyLogic
Source: JMIR Res Protoc. 2017 Jul 17;6(7):e138. doi: 10.2196/resprot.6213 (PMC5537560; doi:10.2196/resprot.6213)
Supplement: Multimedia Appendix 1 [file resprot_v6i7e138_app1.pdf]

APPENDIX

Sample codes

Shapefile database connection string

The correct connection string for connecting to the database of a shapefile depends on the OS and the dBase associated drivers installed on it. In the example here, the OS is a 64-bit Microsoft Windows with a 64-bit Access Database Engine installed on it. If Anylogic is running on a 32-bit Java virtual machine, the required database driver must exist in the 32-bit version of ODBC data sources, and vice-versa for the 64-bit versions. This driver could be either MS Access dBase drive or FoxPro driver. The following Java example code is using the MS Access driver.

```
try {
    Class.forName("sun.jdbc.odbc.JdbcOdbcDriver");
    String connString = "jdbc:odbc:Driver={Microsoft Access dBASE Driver (*.dbf, *.ndx, *.mdx)};
DefaultDir=D:\\";
    // D:\ is the database location
    // Microsoft Access dBASE Driver (*.dbf, *.ndx, *.mdx) exists in Data Sources (ODBC)
    java.sql.Connection conn = java.sql.DriverManager.getConnection(connString);
    String sql="SELECT * from Grid"; // Grid.dbf is the file name
    java.sql.Statement stmt=conn.createStatement();
    java.sql.ResultSet resultSet=stmt.executeQuery(sql);
    while (resultSet!= null & resultSet.next())
        traceln(resultSet.getString(1));

    traceln("Done!");
 }
catch (ClassNotFoundException e)
 {
    e.printStackTrace();
 }
 catch (SQLException e) {
  e.printStackTrace();
 }
```

Another connection string sample for C#.NET is as follows.

```
System.Data.OleDb.OleDbConnection connTest = new System.Data.OleDb.OleDbConnection();
connTest.ConnectionString = @"Provider=Microsoft.Jet.OLEDB.4.0;Data Source=D:\;Extended Properties=dBASE
IV;User ID=Admin;Password=;";
connTest.Open();
OleDbCommand com = new OleDbCommand("SELECT * FROM Grid",connTest);
```

Conversion of shapfile polygon features to Anylogic GIS regions

Polygon features in a shapefile are known as *PoliticalArea(s)* in Anylogic. The code below demonstrates how to extract the coordinates of all political areas in a shapefile, and store them in binary file using the libraries available in Anylogic 7.0.3. First a Java class called *GISPolygon* needs to be defined with the minimal functions. The coordinates of shapefile polygons are then stored in an instance of *GISPolygon*, and is saved as a binary file on the hard disk.

```
java.util.LinkedHashMap <Integer, GISPolygon> ShapefilePolygons = new java.util.LinkedHashMap<Integer,
GISPolygon>();
// Let's assume there's a GIS map component called map, and //
// the first shapefile on the map is the shapefile of our interest //
Object[] politicalAreasObjects =  map.getLayers()[0].getPoliticalAreas().toArray();
PoliticalArea polArea;
GISPolygon gisPolygon;
int featureID=-1; // FID in shapefiles
for (int i=0;i<politicalAreasObjects.length;i++)
{
    polArea = (PoliticalArea) politicalAreasObjects[i];
    featureID = (int) Float.parseFloat(polArea.name); // The name coloumn index of the shapefile on the map
is set to refer to the FID
    OMGeometryList gmList = polArea.getGeometry();
    OMGraphicList grList = (OMGraphicList) gmList;
    Object parent = grList.getOMGraphicAt(0);
    Object child = ((OMGraphicList) parent).getOMGraphicAt(0);
    // There may be a number of nested layers of features in the shapefile//
    while (child.getClass().equals(OMGraphicList.class)) {
        child = ((OMGraphicList) child).getOMGraphicAt(0);
```

```
        parent = ((OMGraphicList) parent).getOMGraphicAt(0);
    }
    int polyCount = ((OMGraphicList) parent).size(); // The number of political areas under the same FID
    gisPolygon = new GISPolygon(featureID);
    for(int j=0;j<polyCount;j++)
    {
        OMPoly poly = (OMPoly) ((OMGraphicList) parent).getOMGraphicAt(j);
        double[] polyCoords =  poly.getLatLonArray();
        ProjMath.arrayRadToDeg(polyCoords);
        gisPolygon.AddLatLonArray(polyCoords);
    }
    ShapefilePolygons.put(featureID, gisPolygon);
}
gisPolygon.SaveShapefilePolgons(ShapefilePolygons, "D:\\MyShapefile.dat"); // Store on hard disk as a binray
file
```

The source code for the *GISPolygon* class is as follows.

```
public class GISPolygon implements Serializable {
    public ArrayList<double[]> latLonArrayList;
    public int npolygons=0;
    public int featureID;
    public GISPolygon() {
        this.featureID = -1; this.npolygons=0;
    }
    public GISPolygon(int featureID) {
        this.npolygons = 0; this.featureID = featureID;
    }
    public void AddLatLonArray(double[] latLonArray) {
        if (this.latLonArrayList == null)
                this.latLonArrayList = new ArrayList<double[]>();

            this.latLonArrayList.add(latLonArray);
            this.npolygons ++;
    }
    public void SaveShapefilePolgons(Object shapefilePolygons, String filePath) {
        try {
            FileOutputStream fout = new FileOutputStream(filePath);
            ObjectOutputStream oos = new ObjectOutputStream(fout);
            oos.writeObject(shapefilePolygons);
            oos.flush(); oos.close();
            System.out.println("File saved!");
        }
        catch(Exception ex) {
            traceln(ex.toString());
        }
    }
    private static final long serialVersionUID = 1L;
}
```

Some functions and libraries used above, such as casting from *OMGeometryList* to *OMGraphicList*, may not be available in all Anylogic versions. However, the coordinate extraction procedure is still similar, and could be adopted by developers.

Finally, the code below shows how to restore saved coordinates, and display them as Anylogic *GISregion(s)* on a map. It is notable that the *GISregion* component is not available in Anylogic 7.0.3, as such the code below was tested in Anylogic 7.2.0 PLE.

```
LinkedHashMap <Integer ,GISPolygon> loadedPolygon = new java.util.LinkedHashMap<Integer, GISPolygon>();
LinkedHashMap <Integer ,GISMultiRegion> MultiGISRegionsList = new LinkedHashMap<Integer, GISMultiRegion>();
try {
    ObjectInputStream in = new ObjectInputStream(new FileInputStream("D:\\MyShapefile.dat"));
    loadedPolygon = (LinkedHashMap<Integer, GISPolygon>) in.readObject();
    in.close();
}
catch(Exception ex) {
    traceln(ex.toString());
}
GISRegion gisRegion;
GISMultiRegion gisMulti;
for (Integer entryKey : loadedPolygon.keySet()){
    GISPolygon poly = loadedPolygon.get(entryKey);
```

```
    gisMulti = new GISMultiRegion(entryKey.toString());
    for (int i=0; i<poly.latLonArrayList.size();i++) {
        gisRegion = new GISRegion(map, poly.latLonArrayList.get(i));
        gisMulti.add(gisRegion);
        map.add(gisRegion);
    }
    MultiGISRegionsList.put(entryKey, gisMulti);
}
```

Birds Species Data

The collected data on bird species can be found in Table I below.

Table I BIRDS SPECIES

Family and Species	Common Name	Home Range [a]	Flight Speed [b]	Roosting [c]	Breeding Months	Sources
**Accipitridae**						
Accipiter cooperii	Cooper's Hawk	905	9	Solitary	4 - 7	[1–3]
Accipiter striatus	Sharp-shinned Hawk	918	7	Solitary	4 - 8	[1,4]
Buteo jamaicensis	Red-tailed Hawk	1163	9	Solitary	2 - 9	[5–8]
Buteo platypterus	Broad-winged Hawk	583	11	Solitary	5 - 8	[9,10]
Buteo regalis	Ferruginous Hawk	1652	16	Solitary	4 - 8	[11–13]
Buteo swainsoni	Swainson's Hawk	2249	7	Communal	4 - 8	[11,14,15]
Circus cyaneus	Northern Harrier	910	9	Solitary	4 - 9	[6,16,17]
Haliaeetus leucocephalus	Bald Eagle	2622	13	Solitary	4 - 9	[18–20]
**Alaudidae**						
Eremophila alpestris	Horned Lark	127	11	Flocking	3 - 8	[21–23]
**Alcedinidae**						
Megaceryle alcyon	Belted Kingfisher	1609	8	Solitary	4 - 8	[24–26]
**Apodidae**						
Chaetura pelagica	Chimney Swift	4000	13	Communal	6 - 8	[27,28]
**Bombycillidae**						
Bombycilla cedrorum	Cedar Waxwing	36	9	Communal	6 - 10	[29–31]
**Calcariidae**						
Calcarius ornatus	Chestnut-collared Longspur	112	9	Flocking	5 - 8	[32,33]
**Caprimulgidae**						
Chordeiles minor	Common Nighthawk	523	10	Flocking	6 - 9	[11,34]
**Cardinalidae**						
Passerina cyanea	Indigo Bunting	160	9	Solitary	6 - 9	[11,35]
Pheucticus ludovicianus	Rose-breasted Grosbeak	140	8	Solitary	6 - 8	[36–38]
Piranga olivacea	Scarlet Tanager	199	8	Solitary	6 - 8	[37,39]
Spiza americana	Dickcissel	390	11	Flocking	5 - 8	[11,40,41]
**Cathartidae**						
Cathartes aura	Turkey Vulture	12657	13	Communal	5 - 9	[42–44]
**Certhiidae**						
Certhia americana	Brown Creeper	500	7	Solitary	5 - 8	[37,45]
**Columbidae**						
Columba livia	Rock Pigeon	5300	16	Communal	4 - 11	[46,47]
Zenaida macroura	Mourning Dove	4000	17	Communal	3 - 10	[27,48,49]
Corvus brachyrhynchos	American Crow	1555	11	Flocking	3 - 6	[50–52]
**Corvidae**						
Corvus corax	Common Raven	3590	11	Communal	3 - 7	[50,53]
Cyanocitta cristata	Blue Jay [d]	103	9	Solitary	4 - 6	[22,54–56]
Perisoreus canadensis	Gray Jay [d]	455	9	Solitary	3 - 6	[55,57]
Pica hudsonia	Black-billed Magpie	126	8	Flocking	4 - 6	[58,59]
**Cuculidae**						
Coccyzus erythropthalmus	Black-billed Cuckoo	305	10	Solitary	6 - 10	[11,60,61]

**Emberizidae**						
Ammodramus bairdii	Baird's Sparrow	200	7	Flocking	6 - 9	[62,63]
Ammodramus leconteii	Le Conte's Sparrow	200	7	Solitary	6 - 9	[62,64]
Ammodramus savannarum	Grasshopper Sparrow	75	7	Solitary	6 - 8	[62,65,66]
Chondestes grammacus	Lark Sparrow	139	12	Flocking	5 - 8	[11,31,67]
Junco hyemalis	Dark-eyed Junco	82	8	Flocking	5 - 9	[26,37,68]
Melospiza georgiana	Swamp Sparrow	113	13	Flocking	5 - 8	[11,69,70]
Melospiza lincolnii	Lincoln's Sparrow	100	13	Solitary	6 - 8	[11,71]
Melospiza melodia	Song Sparrow	113	13	Solitary	3 - 9	[11,72,73]
Passerculus sandwichensis	Savannah Sparrow	170	16	Flocking	6 - 9	[22,74,75]
Pipilo erythrophthalmus	Eastern Towhee	300	8	Solitary	5 - 8	[37,76]
Pipilo maculatus	Spotted Towhee	157	8	Solitary	4 - 8	[37,77–79]
Pooecetes gramineus	Vesper Sparrow	142	7	Flocking	5 - 9	[11,80,81]
Spizella pallida	Clay-colored Sparrow	99	9	Flocking	6 - 8	[11,38,82]
Spizella passerina	Chipping Sparrow	99	9	Flocking	4 - 9	[11,38,73,83]
Zonotrichia albicollis	White-throated Sparrow	93	8	Flocking	6 - 8	[11,84]
**Falconidae**						
Falco columbarius	Merlin	2523	14	Solitary	3 - 9	[85,86]
Falco sparverius	American Kestrel	671	10	Solitary	4 - 8	[6,87,88]
**Fringillidae**						
Carpodacus mexicanus	House Finch	1500	6	Communal	4 - 8	[89–91]
Carpodacus purpureus	Purple Finch	380	6	Solitary	4 - 9	[90,92,93]
Coccothraustes vespertinus	Evening Grosbeak	359	17	Flocking	5 - 8	[94–96]
Loxia curvirostra	Red Crossbill	254	9	Communal	1 - 10	[22,37,70,97,98]
Loxia leucoptera	White-winged Crossbill	1000	9	Communal	1 - 11	[37,99]
Spinus pinus	Pine Siskin	113	15	Communal	4 - 8	[70,100,101]
Spinus tristis	American Goldfinch	800	7	Communal	7 - 9	[22,102,103]
**Hirundinidae**						
Hirundo rustica	Barn Swallow	600	7	Communal	5 - 9	[104–106]
Petrochelidon pyrrhonota	Cliff Swallow	1500	8	Communal	5 - 8	[107,108]
Progne subis	Purple Martin	2871	12	Communal	5 - 8	[22,49,109–111]
Riparia riparia	Bank Swallow	800	14	Communal	4 - 8	[16,106,112]
Stelgidopteryx serripennis	Northern Rough-winged Swallow	500	7	Solitary	5 - 7	[105,113]
Tachycineta bicolor	Tree Swallow	4000	7	Communal	5 - 7	[49,106,114,115]
**Icteridae**						
Agelaius phoeniceus	Red-winged Blackbird	1609	7	Communal	4 - 8	[116–119]
Dolichonyx oryzivorus	Bobolink	90	7	Flocking	6 - 8	[119–121]
Euphagus carolinus	Rusty Blackbird	345	8	Communal	5 - 8	[122]
Euphagus cyanocephalus	Brewer's Blackbird	1600	12	Communal	2 - 8	[123–125]
Icterus galbula	Baltimore Oriole	100	11	Solitary	5 - 7	[11,70,126]
Icterus spurius	Orchard Oriole	113	11	Communal	6 - 8	[11,31,127]
Molothrus ater	Brown-headed Cowbird	1186	13	Communal	4 - 8	[128,129]
Quiscalus quiscula	Common Grackle	12921	14	Communal	4 - 7	[27,116,130,131]
Sturnella neglecta	Western Meadowlark	149	9	Flocking	4 - 9	[70,132]

Xanthocephalus xanthocephalus	Yellow-headed Blackbird	1600	10	Communal	5 - 8	[26,133,134]
**Laniidae**						
Lanius ludovicianus	Loggerhead Shrike	207	13	Solitary	3 - 7	[6,88,135,136]
**Mimidae**						
Dumetella carolinensis	Gray Catbird	37	7	Solitary	5 - 8	[11,70,137]
Mimus polyglottos	Northern Mockingbird	102	8	Solitary	3 - 8	[6,37,70,138,139]
Toxostoma rufum	Brown Thrasher	113	10	Solitary	4 - 8	[37,140,141]
**Motacillidae**						
Anthus spragueii	Sprague's Pipit	143	6	Solitary	5 - 8	[142,143]
**Pandionidae**						
Pandion haliaetus	Osprey	14000	13	Solitary	5 - 9	[27,144]
**Paridae**						
Poecile atricapillus	Black-capped Chickadee	216	5	Communal	4 - 7	[6,145–147]
Poecile hudsonicus	Boreal Chickadee	216	5	Flocking	5 - 9	[146,148,149]
**Parulidae**						
Cardellina canadensis	Canada Warbler	80	7	Flocking	6 - 8	[37,150–152]
Cardellina pusilla	Wilson's Warbler	213	7	Solitary	6 - 8	[37,152–154]
Geothlypis philadelphia	Mourning Warbler [d]	113	7	Solitary	6 - 9	[6,37,70,155,156]
Geothlypis trichas	Common Yellowthroat	96	7	Solitary	6 - 8	[37,70,157,158]
Mniotilta varia	Black-and-white Warbler	145	7	Solitary	5 - 8	[37,152,159,160]
Oporornis agilis	Connecticut Warbler	39	7	Solitary	6 - 8	[37,152,159,161]
Oreothlypis celata	Orange-crowned Warbler	80	7	Solitary	4 - 8	[37,152,162,163]
Oreothlypis peregrina	Tennessee Warbler	124	7	Flocking	7 - 9	[31,37,152,164,165]
Oreothlypis ruficapilla	Nashville Warbler [d]	113	7	Solitary	6 - 8	[37,70,152,166]
Parkesia noveboracensis	Northern Waterthrush	206	8	Solitary	6 - 8	[37,167]
Seiurus aurocapilla	Ovenbird	98	8	Solitary	5 - 8	[6,37,168]
Setophaga americana	Northern Parula	45	7	Solitary	4 - 8	[37,70,169]
Setophaga castanea	Bay-breasted Warbler	106	7	Solitary	6 - 8	[37,70,170]
Setophaga coronata	Yellow-rumped Warbler	169	7	Flocking	6 - 8	[37,171,172]
Setophaga fusca	Blackburnian Warbler	59	7	Flocking	6 - 8	[6,37,173]
Setophaga magnolia	Magnolia Warbler	113	7	Solitary	6 - 9	[6,37,70,174]
Setophaga palmarum	Palm Warbler	110	7	Solitary	5 - 8	[37,175]
Setophaga pensylvanica	Chestnut-sided Warbler	95	7	Flocking	6 - 9	[31,37,176]
Setophaga petechia	Yellow Warbler	490	11	Flocking	6 - 8	[152,177]
Setophaga pinus	Pine Warbler	99	7	Flocking	5 - 7	[37,178,179]
Setophaga ruticilla	American Redstart	45	6	Solitary	6 - 8	[6,37,70,180]
Setophaga striata	Blackpoll Warbler	117	6	Flocking	6 - 8	[37,181,182]
Setophaga tigrina	Cape May Warbler	56	7	Solitary	6 - 8	[37,183]
Setophaga virens	Black-throated Green Warbler	113	7	Flocking	5 - 8	[6,37,70,184]
Vermivora chrysoptera	Golden-winged Warbler	138	7	Solitary	5 - 8	[37,164,185]
**Passeridae**						
Passer domesticus	House Sparrow	1500	13	Communal	4 - 9	[27,49,186,187]
**Phasianidae**						
Bonasa umbellus	Ruffed Grouse	164	8	Solitary	4 - 11	[188,189]

Meleagris gallopavo	Wild Turkey	3586	14	Communal	4 - 11	[22,190]
Perdix perdix	Gray Partridge	623	13	Communal	4 - 7	[191–193]
Phasianus colchicus	Ring-necked Pheasant	339	12	Flocking	4 - 10	[194–196]
Tympanuchus phasianellus	Sharp-tailed Grouse	608	13	Communal	5 - 8	[11,197]
**Picidae**						
Colaptes auratus	Northern Flicker	282	7	Solitary	5 - 7	[198,199]
Dryocopus pileatus	Pileated Woodpecker	896	7	Solitary	5 - 7	[56,70,199,200]
Melanerpes erythrocephalus	Red-headed Woodpecker	164	5	Solitary	5 - 9	[201,202]
Picoides arcticus	Black-backed Woodpecker	594	7	Solitary	6 - 8	[199,201]
Picoides dorsalis	American Three-toed Woodpecker	608	7	Solitary	6 - 8	[199,201,203]
Picoides pubescens	Downy Woodpecker	126	6	Solitary	4 - 7	[199,204]
Picoides villosus	Hairy Woodpecker	460	8	Solitary	4 - 8	[199,205–208]
**Regulidae**						
Regulus calendula	Ruby-crowned Kinglet	138	6	Solitary	5 - 8	[37,209]
Regulus satrapa	Golden-crowned Kinglet	89	6	Solitary	6 - 9	[37,70,210]
**Sittidae**						
Sitta canadensis	Red-breasted Nuthatch	178	5	Solitary	5 - 8	[70,211,212]
Sitta carolinensis	White-breasted Nuthatch	219	5	Solitary	5 - 7	[70,146,213]
**Strigidae**						
Asio flammeus	Short-eared Owl	511	12	Flocking	4 - 7	[11,214,215]
Bubo virginianus	Great Horned Owl	981	17	Solitary	3 - 4	[214,216–218]
Strix varia	Barred Owl	689	6	Solitary	2 - 10	[219,220]
**Sturnidae**						
Sturnus vulgaris	European Starling	7500	11	Communal	4 - 7	[27,49,116,221,222]
**Trochilidae**						
Archilochus colubris	Ruby-throated Hummingbird	183	13	Solitary	5 - 10	[223–226]
**Troglodytidae**						
Cistothorus palustris	Marsh Wren	56	7	Solitary	4 - 9	[227–229]
Cistothorus platensis	Sedge Wren	139	7	Solitary	6 - 9	[37,230–232]
Troglodytes aedon	House Wren	75	7	Solitary	5 - 9	[6,37,233,234]
Troglodytes hiemalis	Winter Wren	138	7	Solitary	5 - 9	[37,235]
**Turdidae**						
Catharus fuscescens	Veery [d]	113	15	Flocking	6 - 7	[70,236,237]
Catharus guttatus	Hermit Thrush	103	15	Communal	5 - 9	[70,237,238]
Catharus ustulatus	Swainson's Thrush	128	15	Solitary	6 - 9	[237,239,240]
Sialia currucoides	Mountain Bluebird	147	8	Flocking	4 - 9	[11,241,242]
Sialia sialis	Eastern Bluebird	247	8	Communal	3 - 9	[6,11,243]
Turdus migratorius	American Robin	400	9	Communal	4 - 8	[27,37,244–247]
**Tyrannidae**						
Contopus cooperi	Olive-sided Flycatcher	219	7	Solitary	6 - 9	[248,249]
Contopus sordidulus	Western Wood-Pewee	82	7	Solitary	5 - 9	[250–252]
Contopus virens	Eastern Wood-Pewee	118	7	Solitary	5 - 9	[38,250,253]
Empidonax alnorum	Alder Flycatcher	98	13	Solitary	6 - 8	[70,254,255]
Empidonax flaviventris	Yellow-bellied Flycatcher	113	13	Flocking	6 - 8	[70,255,256]

Empidonax minimus	Least Flycatcher	60	13	Communal	6 - 8	[6,70,255,257,258]
Empidonax traillii	Willow Flycatcher	81	13	Solitary	6 - 9	[255,259,260]
Myiarchus crinitus	Great Crested Flycatcher	101	10	Solitary	5 - 8	[70,248,255,261]
Sayornis phoebe	Eastern Phoebe [e]	95	10	Solitary	4 - 8	[70,262,263]
Sayornis saya	Say's Phoebe [e]	94	10	Solitary	5 - 8	[264,265]
Tyrannus tyrannus	Eastern Kingbird	212	10	Flocking	6 - 8	[11,38,263,266]
Tyrannus verticalis	Western Kingbird	219	8	Solitary	5 - 7	[11,22,267,268]
**Vireonidae**						
Vireo flavifrons	Yellow-throated Vireo	100	8	Solitary	6 - 8	[37,269]
Vireo gilvus	Warbling Vireo	100	8	Solitary	5 - 8	[37,270–272]
Vireo olivaceus	Red-eyed Vireo	109	8	Flocking	5 - 8	[37,270,273]
Vireo philadelphicus	Philadelphia Vireo	113	8	Solitary	6 - 8	[37,274]
Vireo solitarius	Blue-headed Vireo	100	8	Solitary	6 - 8	[37,275]

[a.] Home range values are the ceiling for the average radius of an estimated circular home range area in meters.

[b.] Flight speed values are reported in meters per second. As it was not clear as to the type of speed that different papers reported, it is not recommended to compare the birds' speed by these values.

[c.] Flocking under the Roosting column means that the species roost individually or in pairs during the breeding season, and then form flocks for migration in the fall.

[d.] It is notable that for the Mourning Warbler, Veery, and Nashville Warbler, the sources of data were not clear as to the type of roosting that they did. The entries given represent a best guess, but further data would be needed for greater precision. Specifically, for the Veery, no mention of communal roosting was found in literature, but no mention of any other type was found. The Blue Jay and Gray Jay were difficult to properly categorize due to ambiguity in literature regarding their roosting behaviour.

[e.] For the flight speeds of Eastern Phoebe and Say's Phoebe species, the average speed of the Tyrannidae birds in the dataset is used.

References for Appendix

1. Goodwin MBB V. Flight-Speeds of Hawks and Crows. Auk [Internet] 1943 [cited 2015 Nov 5];60(4):487–492. Available from: https://sora.unm.edu/node/18587
2. Curtis OE, Rosenfield RN, Bielefeldt. J. Cooper's Hawk (Accipiter cooperii). Poole A, Gill F, editors. Birds North Am Online [Internet] 2006; Available from: http://bna.birds.cornell.edu/BNA/account/Coopers_Hawk/RECOMMENDED_CITATION.html
3. Polite C, Kiff L. COOPER'S HAWK Accipiter cooperii. In: Zeiner DC, Jr. WFL, Mayer KE, White M, editors. Calif Wildl Habitat Relationships Vol I-III. Sacramento, California: California Depart. of Fish and Game; 1990.
4. Bildstein KL, Meyer K. Sharp-shinned Hawk (Accipiter striatus). Poole A, Gill F, editors. Birds North Am Online [Internet] 2000; Available from: http://bna.birds.cornell.edu/bna/species/482
5. Johnson S. Red-Tailed Hawks (Buteo jamaicensis) [Internet]. 2011 [cited 2015 Nov 5]. Available from: http://beautyofbirds.com/redtailedhawks.html. Arhived at: http://www.webcitation.org/6lCvnOQPe
6. Schoener TW. Sizes of Feeding Territories among Birds Ecological Society of America. Ecol Soc Am Stable 1968;49(1):123–141.
7. Polite C, Pratt J, Bailey S. RED-TAILED HAWK Buteo jamaicensis. In: Zeiner DC, Jr. WFL, Mayer KE, White M, editors. Calif Wildl Habitat Relationships Vol I-III. Sacramento, California: California Depart. of Fish and Game; 1990.
8. Preston CR, Beane RD. Red-tailed Hawk (Buteo jamaicensis). Poole A, Gill F, editors. Birds North Am Online [Internet] 2009; Available from: http://bna.birds.cornell.edu/BNA/account/Red-tailed_Hawk/RECOMMENDED_CITATION.html
9. Goodrich LJ, Crocoll ST, Senner SE. Broad-winged Hawk (Buteo platypterus). Poole A, Gill F, editors. Birds North Am Online [Internet] 2014; Available from: http://bna.birds.cornell.edu/BNA/account/Broad-winged_Hawk/RECOMMENDED_CITATION.html
10. Bloom PH, Mccrary MD, Gibson MJ. Red-Shouldered Hawk Home-Range and Habitat Use in Southern California. J Wildl Manage [Internet] 1993;57(2):258–265. Available from: http://www.jstor.org/stable/3809422
11. Bumstead P. Canadian Feathers : a Loon-atics Guide to Anting, Mimicry and Dump-nesting [Internet]. Simply Wild Publications; 2001 [cited 2015 Nov 5]. Available from: https://books.google.com/books?id=vmkmuyvhxqAC&pgis=1 ISBN:0968927807
12. Dwight GS, Murphy JR. Breeding ecology of raptors in the eastern Great Basin of Utah [Internet]. Brigham Young Univ Sci Bull - Biol Ser. 1973 [cited 2015 Nov 5]. p. 1–76. Available from: https://ojs.lib.byu.edu/spc/index.php/BYUSciBullBioS/article/view/30664

13. Bechard MJ, Schmutz JK. Ferruginous Hawk (Buteo regalis). Poole A, Gill F, editors. Birds North Am Online [Internet] 1995; Available from: http://bna.birds.cornell.edu/bna/species/172

14. Bechard MJ, Houston CS, Sarasola JH, England AS. Swainson's Hawk (Buteo swainsoni). Poole A, Gill F, editors. Birds North Am Online [Internet] 2010; Available from: http://bna.birds.cornell.edu/BNA/account/Swainsons_Hawk/RECOMMENDED_CITATION.html

15. Polite C, Kiff L. SWAINSON'S HAWK Buteo swainsoni. In: Zeiner DC, Jr. WFL, Mayer KE, White M, editors. Calif Wildl Habitat Relationships Vol I-III. Sacramento, California: California Depart. of Fish and Game; 1990.

16. Alerstam T, Rosén M, Bäckman J, Ericson PGP, Hellgren O. Flight Speeds among Bird Species: Allometric and Phylogenetic Effects. Sheldon B, editor. PLoS Biol [Internet] 2007 Jul 17;5(8):e197. Available from: http://dx.plos.org/10.1371/journal.pbio.0050197 DOI:10.1371/journal.pbio.0050197

17. Smith KG, Wittenberg SR, Macwhirter RB, Bildstein KL. Northern Harrier (Circus cyaneus). Poole A, Gill F, editors. Birds North Am Online [Internet] 2011; Available from: http://bna.birds.cornell.edu/BNA/account/Northern_Harrier/RECOMMENDED_CITATION.html

18. Travsky A, Equvais DGP. Species Assessment for Bald Eagle (Haliaeetus leucocephalus) in Wyoming [Internet]. 2004. Available from: http://www.blm.gov/pgdata/etc/medialib/blm/wy/wildlife/animal-assessmnts.Par.41209.File.dat/BaldEagle.pdf. Archived at: http://www.webcitation.org/6lCxhG1lp

19. Rutledge H. Bald Eagle Description Page 2 [Internet]. Am Bald Eagle Inf. [cited 2015 Nov 5]. Available from: http://www.baldeagleinfo.com/eagle/eagle8.html Archived at: http://www.webcitation.org/6lD5OKOFU

20. Buehler DA. Bald Eagle (Haliaeetus leucocephalus). Poole A, Gill F, editors. Birds North Am Online [Internet] 2000; Available from: http://bna.birds.cornell.edu/BNA/account/Bald_Eagle/RECOMMENDED_CITATION.html

21. Cannings RJ, Threlfall W. Horned Lark Breeding Biology at Cape St. Mary's, Newfoundland. Wilson Bull [Internet] 1981 [cited 2015 Nov 19];93(4):12. Available from: http://www.jstor.org/stable/4161544

22. Cooke MT. Flight speed of birds [Internet]. Washington, D.C. : U.S. Dept. of Agriculture,; 1937 [cited 2015 Nov 4]. Available from: http://www.biodiversitylibrary.org/bibliography/64128 DOI:10.5962/bhl.title.64128

23. Beason RC. Horned Lark (Eremophila alpestris). Poole A, Gill F, editors. Birds North Am Online [Internet] 1995; Available from: http://bna.birds.cornell.edu/BNA/account/Horned_Lark/RECOMMENDED_CITATION.html

24. Kelly JF, Bridge ES, Hamas MJ. Belted Kingfisher (Ceryle alcyon). Poole A, Gill F, editors. Birds North Am Online [Internet] 2009; Available from: http://bna.birds.cornell.edu/BNA/account/Belted_Kingfisher/RECOMMENDED_CITATION.html

25. Green M, Mewaldt L, Duke R, Winkler D. BELTED KINGFISHER Megaceryle alcyon. In: Zeiner DC, Jr. WFL, Mayer KE, White M, editors. Calif Wildl Habitat Relationships Vol I-III. Sacramento, California: California Depart. of Fish and Game; 1990.

26. Wood HB. Flight Speed of Some Birds. Auk [Internet] 1933 [cited 2015 Nov 4];50(4):452–453. Available from: https://sora.unm.edu/node/16252 DOI:10.2307/4077639

27. Schnell GD, Hellack JJ. Flight Speeds of Brown Pelicans, Chimney Swifts, and Other Birds. Bird-Banding 1978;49(2):108–112.

28. Steeves TK, Kearney-McGee SB, Rubega MA, Cink CL, Collins CT. Chimney Swift (Chaetura pelagica). Poole A, Gill F, editors. Birds North Am Online [Internet] 2014; Available from: http://bna.birds.cornell.edu/bna/species/646

29. Witmer MC, Mountjoy DJ, Elliot L. Cedar Waxwing (Bombycilla cedrorum). Poole A, Gill F, editors. Birds North Am Online [Internet] 2014; Available from: http://bna.birds.cornell.edu/BNA/account/Cedar_Waxwing/RECOMMENDED_CITATION.html

30. Khanna DR. Biology of Birds [Internet]. Discovery Publishing House; 2005 [cited 2015 Nov 13]. Available from: https://books.google.com/books?id=fDblIChi7KwC&pgis=1 ISBN:817141933X

31. Gillihan SW. Bird Conservation on Golf Courses: A Design and Management Manual [Internet]. John Wiley & Sons; 2000 [cited 2015 Nov 2]. Available from: https://books.google.com/books?id=8qOv_8kRyJgC&pgis=1 ISBN:1575041138

32. Hussell DJ, Montgomerie R. Lapland Longspur (Calcarius lapponicus). Poole A, Gill F, editors. Birds North Am Online [Internet] 2002; Available from: http://bna.birds.cornell.edu/BNA/account/Lapland_Longspur/RECOMMENDED_CITATION.html

33. Bleho B, Ellison K, Hill DP, Gould LK. Chestnut-collared Longspur (Calcarius ornatus). Poole A, Gill F, editors. Birds North Am Online [Internet] 2015; Available from: http://bna.birds.cornell.edu/bna/species/288

34. Brigham RM, Ng J, Poulin RG, Grindal SD. Common Nighthawk (Chordeiles minor). Poole A, Gill F, editors. Birds North Am Online [Internet] 2011; Available from: http://bna.birds.cornell.edu/BNA/account/Common_Nighthawk/RECOMMENDED_CITATION.html

35. Payne RB. Indigo Bunting (Passerina cyanea). Poole A, Stettenheim P, Gill F, editors. Birds North Am Online [Internet] 2006;(4). Available from: http://bna.birds.cornell.edu/BNA/account/Indigo_Bunting/RECOMMENDED_CITATION.html

36. Wyatt VE, Francis CM. Rose-breasted Grosbeak (Pheucticus ludovicianus). Poole A, Gill F, editors. Birds North Am Online [Internet] 2002; Available from: http://bna.birds.cornell.edu/BNA/account/Rose-breasted_Grosbeak/RECOMMENDED_CITATION.html

37. Cabrera-Cruz SA, Mabee TJ, Patraca RV. Using Theoretical Flight Speeds to Discriminate Birds from Insects in Radar Studies. Condor [Internet] 2013 May;115(2):263–272. Available from: http://www.bioone.org/doi/abs/10.1525/cond.2013.110181 DOI:10.1525/cond.2013.110181

38. Odum EP, Kuenzler EJ. Measurement of Territory and Home Range Size in Birds. Auk [Internet] 1955 Apr [cited 2015 Nov 7];72(2):128–137. Available

from: http://www.jstor.org/stable/info/10.2307/4081419

39. Mowbray TB. Scarlet Tanager (Piranga olivacea). Poole A, Gill F, editors. Birds North Am Online [Internet] 1999; Available from: http://bna.birds.cornell.edu/BNA/account/Scarlet_Tanager/RECOMMENDED_CITATION.html

40. Temple S. Dickcissel (Spiza americana). Poole A, Gill F, editors. Birds North Am Online [Internet] 2002; Available from: http://bna.birds.cornell.edu/BNA/account/Dickcissel/RECOMMENDED_CITATION.html

41. Wells KMS, Millspaugh JJ, Ryan MR, Hubbard MW. Factors Affecting Home Range Size and Movements of Post-Fledging Grassland Birds. Wilson J Ornithol [Internet] The Wilson Ornithological Society; 2008 Mar [cited 2015 Nov 27];120(1):120–130. Available from: http://dx.doi.org/10.1676/06-117.1 DOI:10.1676/06-117.1

42. Buckley NJ. Food finding and the influence of information, local enhancement, and communal roosting on foraging success of North American vultures. Auk [Internet] 1996;113(2):473–488. Available from: http://www.jstor.org/stable/4088913 DOI:10.2307/4088913

43. Houston CS, Holroyd GL, Terry B, Blom M, Stoffel MJ. Tracking Saskatchewan Nestling Turkey Vultures. Blue Jay [Internet] 2007;65(4):201–207. Available from: http://www.naturesask.ca/rsu_docs/tracking-saskatchewan-nestling-tv.pdf Archived at: http://www.webcitation.org/6lCxk17k4

44. Kirk DA, Mossman MJ. Turkey Vulture (Cathartes aura). Poole A, Gill F, editors. Birds North Am Online [Internet] 1998; Available from: http://bna.birds.cornell.edu/bna/species/339

45. Poulin J-F, D'Astous E, Villard M-A, Hejl SJ, Newlon KR, Mcfadzen ME, et al. Brown Creeper (Certhia americana). Poole A, Gill F, editors. Birds North Am Online [Internet] 2013; Available from: http://bna.birds.cornell.edu/BNA/account/Brown_Creeper/RECOMMENDED_CITATION.html

46. Lowther PE, Johnston RF. Rock Pigeon (Columba livia). Poole A, Stettenheim P, Gill F, editors. Birds North Am Online [Internet] 2013; Available from: http://bna.birds.cornell.edu/BNA/account/Rock_Pigeon/RECOMMENDED_CITATION.html

47. Baldaccini NE, Giunchi D, Mongini E, Ragionieri L. Foraging flights of wild rock doves (Columba l. livia) : a spatio-temporal analysis. Ital J Zool [Internet] 2000;67(4):371–377. Available from: http://www.tandfonline.com/doi/abs/10.1080/11250000009356342 DOI:10.1080/11250000009356342

48. Otis DL, Schulz JH, Miller D, Mirarchi RE, Baskett TS. Mourning Dove (Zenaida macroura). Poole A, Gill F, editors. Birds North Am Online [Internet] 2008; Available from: http://bna.birds.cornell.edu/BNA/account/Mourning_Dove/RECOMMENDED_CITATION.html

49. Caccamise DF, Fischl J. Patterns of Association of Secondary Species in Roosts of European Starlings and Common Grackles. Wilson Bull [Internet] 1985;97(2):173–182. Available from: http://www.jstor.org/stable/4162069

50. Marzluff JM, Angell T. In the Company of Crows and Ravens [Internet]. Yale University Press; 2008 [cited 2015 Nov 5]. Available from: https://books.google.com/books?id=Bc5YO5PnPmMC&pgis=1 ISBN:0300135262

51. Ward MP, Raim A, Yaremych-Hamer S, Lampman R, Novak RJ. Does the roosting behavior of birds affect transmission dynamics of West Nile virus? Am J Trop Med Hyg [Internet] 2006 Aug [cited 2015 Apr 21];75(2):350–5. Available from: http://www.ncbi.nlm.nih.gov/pubmed/16896147 PMID: 16896147

52. Verbeek NA, Caffrey C. American Crow (Corvus brachyrhynchos). Poole A, Gill F, editors. Birds North Am Online [Internet] 2002; Available from: http://bna.birds.cornell.edu/bna/species/647

53. Boarman WI, Heinrich B. Common Raven (Corvus corax). Poole A, Gill F, editors. Birds North Am Online [Internet] 1999; Available from: http://bna.birds.cornell.edu/BNA/account/Common_Raven/RECOMMENDED_CITATION.html

54. Smith KG, Tarvin KA, Woolfenden GE. Blue Jay (Cyanocitta cristata). Poole A, Gill F, editors. Birds North Am Online [Internet] 2013; Available from: http://bna.birds.cornell.edu/BNA/account/Blue_Jay/RECOMMENDED_CITATION.html

55. Texas Parks and Wildlife Department. Animal Speeds [Internet]. [cited 2015 Nov 6]. Available from: http://tpwd.texas.gov/publications/nonpwdpubs/young_naturalist/animals/animal_speeds/index.phtml. Archived at: http://www.webcitation.org/6lCxGHXZ7

56. Bowman J. Is dispersal distance of birds proportional to territory size? Can J Zool [Internet] 2003 Feb;81(2):195–202. Available from: http://www.nrcresearchpress.com/doi/abs/10.1139/z02-237 DOI:10.1139/z02-237

57. Strickland D, Ouellet H. Gray Jay (Perisoreus canadensis). Poole A, Stettenheim P, Gill F, editors. Birds North Am Online [Internet] 2011; Available from: http://bna.birds.cornell.edu/BNA/account/Gray_Jay/RECOMMENDED_CITATION.html

58. Tobalske BW, Olson NE, Dial KP. Flight style of the black-billed magpie: variation in wing kinematics, neuromuscular control, and muscle composition. J Exp Zool [Internet] 1997 Nov 1 [cited 2015 Nov 27];279(4):313–29. Available from: http://www.ncbi.nlm.nih.gov/pubmed/9360313 PMID: 9360313

59. Trost CH. Black-billed Magpie (Pica hudsonia). Poole A, Gill F, editors. Birds North Am Online [Internet] 1999; Available from: http://bna.birds.cornell.edu/BNA/account/Black-billed_Magpie/RECOMMENDED_CITATION.html

60. Hughes JM. Yellow-billed Cuckoo (Coccyzus americanus). Poole A, Gill F, editors. Birds North Am Online [Internet] 2015; Available from: http://bna.birds.cornell.edu/BNA/account/Yellow-billed_Cuckoo/RECOMMENDED_CITATION.html

61. Hughes JM. Black-billed Cuckoo (Coccyzus erythropthalmus). Poole A, Gill F, editors. Birds North Am Online [Internet] 2001; Available from: http://bna.birds.cornell.edu/BNA/account/Black-billed_Cuckoo/RECOMMENDED_CITATION.html

62. Lowther PE 2005. Le Conte's Sparrow (Ammodramus leconteii). Poole A, editor. Birds North Am Online [Internet] 2005; Available from:

http://bna.birds.cornell.edu/BNA/account/Le_Contes_Sparrow/RECOMMENDED_CITATION.html

63. Green MT, Lowther PE, Jones SL, Davis SK, Dale BC. Baird's Sparrow (Ammodramus bairdii). Poole A, Gill F, editors. Birds North Am Online [Internet] 2002; Available from: http://bna.birds.cornell.edu/BNA/account/Bairds_Sparrow/RECOMMENDED_CITATION.html

64. Baldwin HQ, Jeske CW, Powell MA, Chadwick PC, Barrow WC. Home-Range Size and Site Tenacity of Overwintering Le Conte's Sparrows in a Fire Managed Prairie. Wilson J Ornithol [Internet] 2010 Mar [cited 2015 Nov 6];122(1):139–145. Available from: http://www.bioone.org/doi/abs/10.1676/08-160.1 DOI:10.1676/08-160.1

65. Delany MF., Moore CT., Hamblen JM. Florida grasshopper sparrow management needs [Internet]. Final Rep to Florida Game Fresh Water Fish Comm Tallahassee, Florida. 1992 [cited 2015 Nov 6]. Available from: http://s3.amazonaws.com/file-storage.INDIVIDUAL-ACTIVITIES-CooperativeResearchUnits.digitalmeasures.usgs.edu/cmoore/tech_publications/Delany et al (1992) FGS Final Report-1.pdf. Archived at: http://www.webcitation.org/6lCxp46eH

66. Vickery PD. Grasshopper Sparrow (Ammodramus savannarum). Poole A, Gill F, editors. Birds North Am Online [Internet] 1996; Available from: http://bna.birds.cornell.edu/BNA/account/Grasshopper_Sparrow/RECOMMENDED_CITATION.html

67. Martin JW, Parrish JR. Lark Sparrow (Chondestes grammacus). Poole A, Gill F, editors. Birds North Am Online [Internet] 2000; Available from: http://bna.birds.cornell.edu/BNA/account/Lark_Sparrow/RECOMMENDED_CITATION.html

68. Nolan, Jr. V, Ketterson ED, Cristol DA, Rogers CM, Clotfelter ED, Titus RC, et al. Dark-eyed Junco (Junco hyemalis). Poole A, Gill F, editors. Birds North Am Online [Internet] 2002; Available from: http://bna.birds.cornell.edu/BNA/account/Dark-eyed_Junco/RECOMMENDED_CITATION.html

69. Mowbray TB. Swamp Sparrow (Melospiza georgiana). Poole A, Gill F, editors. Birds North Am Online [Internet] 1997; Available from: http://bna.birds.cornell.edu/BNA/account/Swamp_Sparrow/RECOMMENDED_CITATION.html

70. DeGraaf RM. Technical Guide to Forest Wildlife Habitat Management in New England [Internet]. UPNE; 2006 [cited 2015 Nov 2]. Available from: https://books.google.com/books?id=hwsl9mWPNS4C&pgis=1 ISBN:1584655879

71. Ammon EM. Lincoln's Sparrow (Melospiza lincolnii). Poole A, Gill F, editors. Birds North Am Online [Internet] 1995; Available from: http://bna.birds.cornell.edu/BNA/account/Lincolns_Sparrow/RECOMMENDED_CITATION.html

72. Arcese P, Sogge MK, Marr AB, Patten MA. Song Sparrow (Melospiza melodia). Poole A, Gill F, editors. Birds North Am Online [Internet] 2002; Available from: http://bna.birds.cornell.edu/BNA/account/Song_Sparrow/RECOMMENDED_CITATION.html

73. DeGraaf RM, Yamasaki M. New England Wildlife: Habitat, Natural History, and Distribution [Internet]. UPNE; 2001 [cited 2015 Nov 7]. Available from: https://books.google.com/books?id=mOewbhHSET4C&pgis=1 ISBN:0874519578

74. Ginter DL, Desmond MJ. Influence of foraging and roosting behavior on home-range size and movement patterns of Savannah Sparrows wintering in south Texas. Wilson Bull [Internet] The Wilson Ornithological Society; 2005 Mar [cited 2015 Nov 7];117(1):63–71. Available from: http://www.bioone.org/doi/abs/10.1676/04-017 DOI:10.1676/04-017

75. Wheelwright NT, Rising JD. Savannah Sparrow (Passerculus sandwichensis). Poole A, Gill F, editors. Birds North Am Online [Internet] 2008; Available from: http://bna.birds.cornell.edu/BNA/account/Savannah_Sparrow/RECOMMENDED_CITATION.html

76. Greenlaw JS. Eastern Towhee (Pipilo erythrophthalmus). Poole A, Gill F, editors. Birds North Am Online [Internet] 2015; Available from: http://bna.birds.cornell.edu/BNA/account/Eastern_Towhee/RECOMMENDED_CITATION.html

77. Bartos Smith S, Greenlaw JS. Spotted Towhee (Pipilo maculatus). Poole A, Gill F, editors. Birds North Am Online [Internet] 2015; Available from: http://bna.birds.cornell.edu/BNA/account/Spotted_Towhee/RECOMMENDED_CITATION.html

78. Dobkin D, Mewaldt L, Duke R, Granholm S. SPOTTED TOWHEE Pipilo maculatus. In: Zeiner DC, Jr. WFL, Mayer KE, White M, editors. Calif Wildl Habitat Relationships Vol I-III. Sacramento, California: California Depart. of Fish and Game; 1990.

79. Baker MC, Mewaldt LR. The use of space by white-crowned sparrows: Juvenile and adult ranging patterns and home range versus body size comparisons in an avian granivore community. Behav Ecol Sociobiol [Internet] 1979;6(1):45–52. Available from: http://link.springer.com/article/10.1007/BF00293244 DOI:10.1007/BF00293244

80. Macías-Duarte A, Panjabi AO. Home range and habitat use of wintering Vesper Sparrows in grasslands of the Chihuahuan Desert in Mexico. Wilson J Ornithol [Internet] The Wilson Ornithological Society; 2013 Dec [cited 2015 Nov 7];125(4):755–762. Available from: http://dx.doi.org/10.1676/13-043.1

81. Jones SL, Cornely JE. Vesper Sparrow (Pooecetes gramineus). Poole A, Gill F, editors. Birds North Am Online [Internet] 2002; Available from: http://bna.birds.cornell.edu/BNA/account/Vesper_Sparrow/RECOMMENDED_CITATION.html

82. Grant TA, Knapton RW. Clay-colored Sparrow (Spizella pallida). Poole A, Gill F, editors. Birds North Am Online [Internet] 2012; Available from: http://bna.birds.cornell.edu/BNA/account/Clay-colored_Sparrow/RECOMMENDED_CITATION.html

83. Middleton AL. Chipping Sparrow (Spizella passerina). Poole A, Gill F, editors. Birds North Am Online [Internet] 1998; Available from: http://bna.birds.cornell.edu/BNA/account/Chipping_Sparrow/RECOMMENDED_CITATION.html

84. Falls JB, Kopachena JG. White-throated Sparrow (Zonotrichia albicollis). Poole A, Gill F, editors. Birds North Am Online [Internet] 2010; Available from: http://bna.birds.cornell.edu/BNA/account/White-throated_Sparrow/RECOMMENDED_CITATION.html

85. Becker DM, Sieg CH. Home range and habitat utilization of breeding male Merlins, Falco columbarius, in southeastern Montana. Can Field-Naturalist [Internet] 1987;101:398–403. Available from: http://www.fs.fed.us/rm/pubs_other/rmrs_1987_sieg_c002.pdf

86. Warkentin IG, Sodhi NS, Espie RHM, Poole AF, Oliphant LW, James PC. Merlin (Falco columbarius). Poole A, editor. Birds North Am Online [Internet] 2005; Available from: http://bna.birds.cornell.edu/BNA/account/Merlin/RECOMMENDED_CITATION.html

87. Smallwood JA, Bird DM. American Kestrel (Falco sparverius). Poole A, Gill F, editors. Birds North Am Online [Internet] 2002; Available from: http://bna.birds.cornell.edu/BNA/account/American_Kestrel/RECOMMENDED_CITATION.html

88. Wauer R. How Fast Do Birds Fly? [Internet]. Nat Writ Texas. 2007 [cited 2015 Nov 5]. Available from: http://texasnature.blogspot.ca/2007/02/how-fast-do-birds-fly-by-ro-wauer-on.html. Archived at: http://www.webcitation.org/6lD0TD9vl

89. Dhondt AA, Driscoll MJL, Swarthout ECH. House Finch Carpodacus mexicanus roosting behaviour during the non-breeding season and possible effects of mycoplasmal conjunctivitis. Ibis (Lond 1859) [Internet] 2007;149(1):1–9. Available from: http://doi.wiley.com/10.1111/j.1474-919X.2006.00588.x DOI:10.1111/j.1474-919x.2006.00588.x

90. Tobalske B, Peacock W, Dial K. Kinematics of flap-bounding flight in the zebra finch over a wide range of speeds. J Exp Biol [Internet] 1999 Jul [cited 2015 Nov 6];202 (Pt 13:1725–39. Available from: http://www.ncbi.nlm.nih.gov/pubmed/10359676 PMID: 10359676

91. Badyaev A V., Belloni V, Hill GE. House Finch (Carpodacus mexicanus). Poole A, Gill F, editors. Birds North Am Online [Internet] 2012; Available from: http://bna.birds.cornell.edu/BNA/account/House_Finch/RECOMMENDED_CITATION.html

92. Herman J, McGarry M. Examination of home range in male purple finches (Carpodacus purpureus) [Internet]. Itasca Biol Stn Student Pap. 2011. Available from: http://hdl.handle.net/11299/99539

93. Wootton JT. Purple Finch (Carpodacus purpureus). Poole A, Gill F, editors. Birds North Am Online [Internet] 1996; Available from: http://bna.birds.cornell.edu/BNA/account/Purple_Finch/RECOMMENDED_CITATION.html

94. Gillihan SW, Byers B. Evening Grosbeak (Coccothraustes vespertinus). Poole A, Gill F, editors. Birds North Am Online [Internet] 2001; Available from: http://bna.birds.cornell.edu/BNA/account/Evening_Grosbeak/RECOMMENDED_CITATION.html

95. Dobkin D, Granholm S, Mewaldt L, Duke R. EVENING GROSBEAK Coccothraustes vespertinus. In: Zeiner DC, Jr. WFL, Mayer KE, White M, editors. Calif Wildl Habitat Relationships Vol I-III. Sacramento, California: California Depart. of Fish and Game; 1990.

96. Meinertzhagen R. Speed and Altitude of Bird Flight (With Notes on Other Animals). Ibis (Lond 1859) [Internet] 2008 Apr 3;97(1):81–117. Available from: http://doi.wiley.com/10.1111/j.1474-919X.1955.tb03020.x DOI:10.1111/j.1474-919X.1955.tb03020.x

97. Adkisson CS. Red Crossbill (Loxia curvirostra). Poole A, Gill F, editors. Birds North Am Online [Internet] 1996; Available from: http://bna.birds.cornell.edu/BNA/account/Red_Crossbill/RECOMMENDED_CITATION.html

98. Ellegren H. Speed of migration and migratory flight lengths of passerine birds ringed during autumn migration in Sweden. Ornis Scand 1993;24(3):220–228.

99. Benkman CW. White-winged Crossbill (Loxia leucoptera). Poole A, Stettenheim P, Gill F, editors. Birds North Am Online [Internet] 2012; Available from: http://dx.doi.org.uml.idm.oclc.org/10.2173/bna.27

100. Dawson WR. Pine Siskin (Carduelis pinus). Poole A, Gill F, editors. Birds North Am Online [Internet] 2014; Available from: http://bna.birds.cornell.edu/BNA/account/Pine_Siskin/RECOMMENDED_CITATION.html

101. Videler JJ. Avian Flight [Internet]. Evolution (N Y). 2005. Available from: http://books.google.com/books?id=5Xr9NZdgzP0C&pgis=1 ISBN:0199299927, 9780199299928

102. Coutlee EL. Agonistic Behavior in the American Goldfinch. Wilson Bull [Internet] 1967;79(1):89–109. Available from: https://sora.unm.edu/sites/default/files/journals/wilson/v079n01/p0089-p0109.pdf

103. McGraw KJ, Middleton AL. American Goldfinch (Carduelis tristis). Poole A, Gill F, editors. Birds North Am Online [Internet] 2009; Available from: http://bna.birds.cornell.edu/BNA/account/American_Goldfinch/RECOMMENDED_CITATION.html

104. Brown C, Brown MB. Barn Swallow (Hirundo rustica) [Internet]. Birds North Am Online. 1999. Available from: http://bna.birds.cornell.edu/bna/species/452/articles/introduction

105. Blake RW, Kolotylo R, Cueva H de la. Flight speeds of the barn swallow, Hirundo rustica. Can J Zool [Internet] NRC Research Press Ottawa, Canada; 1990 Jan 14 [cited 2015 Nov 6];68(1):1–5. Available from: http://www.nrcresearchpress.com/doi/abs/10.1139/z90-001 DOI:10.1139/z90-001

106. Heagy A, Badzinski D, Bradley D, Falconer M, McCracken J, Reid RA, et al. Recovery Strategy for the Barn Swallow (Hirundo rustica) in Ontario [Internet]. Peterborough, Ontario; 2014. Available from: http://files.ontario.ca/environment-and-energy/species-at-risk/mnr_sar_rs_brn_swl_en.pdf. Archived at: http://www.webcitation.org/6lCy1x6eO

107. Shelton RM, Jackson BE, Hedrick TL. The mechanics and behavior of cliff swallows during tandem flights. J Exp Biol [Internet] 2014 Aug 1;217(15):2717–2725. Available from: http://jeb.biologists.org/cgi/doi/10.1242/jeb.101329 DOI:10.1242/jeb.101329

108. Brown CR, Brown MB. Cliff Swallow (Petrochelidon pyrrhonota). Poole A, Gill F, editors. Birds North Am Online [Internet] 1995; Available from: http://bna.birds.cornell.edu/BNA/account/Cliff_Swallow/RECOMMENDED_CITATION.html

109. Russell KR, Gauthreaux S a. Spatial and temporal dynamics of a Purple Martin pre-migratory roost. Wilson Bull [Internet] 1999;111(3):354–362.

Available from: http://www.jstor.org/stable/4164099

110. Purple Martin Conservation Association. Purple Martin Terminology [Internet]. [cited 2016 Jan 23]. Available from: https://www.purplemartin.org/purple-martins/biology/41/terminology/ Archived at: http://www.webcitation.org/6lD1ZVpja

111. Tarof S, Brown CR. Purple Martin (Progne subis). Poole A, Gill F, editors. Birds North Am Online [Internet] 2013; Available from: http://bna.birds.cornell.edu/bna/species/287

112. Garrison BA 1999. Bank Swallow (Riparia riparia). Poole A, Gill F, editors. Birds North Am Online [Internet] 1999; Available from: http://bna.birds.cornell.edu/BNA/account/Bank_Swallow/RECOMMENDED_CITATION.html

113. De Jong MJ. Northern Rough-winged Swallow (Stelgidopteryx serripennis). Poole A, Gill F, editors. Birds North Am Online [Internet] 1996; Available from: http://bna.birds.cornell.edu/BNA/account/Northern_Rough-winged_Swallow/RECOMMENDED_CITATION.html

114. Winkler DW, Hallinger KK, Ardia DR, Robertson RJ, Stutchbury BJ, Cohen RR. Tree Swallow (Tachycineta bicolor). Poole A, Stettenheim P, Gill F, editors. Birds North Am Online [Internet] 2011; Available from: http://bna.birds.cornell.edu/BNA/account/Tree_Swallow/RECOMMENDED_CITATION.html

115. Kolotylo RA. Flight Speeds and Energetics of Seven Bird Species [Internet] [M.ScThesis]. University of British Columbia; 1989. Available from: https://circle.ubc.ca/bitstream/id/92363/UBC_1989_A6_7 Archived at: http://www.webcitation.org/6lD1sFkkh

116. Meanley B. The Roosting Behavior of the Red-Winged Black-Bird in the Southern United States. Wilson Bull [Internet] 1965;77(3):218–228. Available from: http://sora.unm.edu/sites/default/files/journals/wilson/v077n03/p0217-p0228.pdf

117. Illinois Natural History Survey. Red-winged blackbird [Internet]. [cited 2015 Nov 6]. Available from: http://wwx.inhs.illinois.edu/collections/birds/ilbirds/56/ Archived at: http://www.webcitation.org/6lD21SkZV

118. Yasukawa K, Searcy WA. Red-winged Blackbird (Agelaius phoeniceus). Poole A, Gill F, editors. Birds North Am Online [Internet] 1995; Available from: http://bna.birds.cornell.edu/BNA/account/Red-winged_Blackbird/RECOMMENDED_CITATION.html

119. Pettingill OS. Ornithology in Laboratory and Field [Internet]. Elsevier; 2013 [cited 2015 Nov 6]. Available from: https://books.google.com/books?id=livLBAAAQBAJ&pgis=1 ISBN:1483263118

120. Renfrew R, Strong AM, Perlut NG, Martin SG, Gavin TA. Bobolink (Dolichonyx oryzivorus). Poole A, Gill F, editors. Birds North Am Online [Internet] 2015; Available from: http://bna.birds.cornell.edu/bna/species/176

121. Wiens JA. An Approach to the Study of Ecological Relationships among Grassland Birds. Ornithol Monogr [Internet] 1969 Jan [cited 2015 Nov 19]; (8):1–93. Available from: http://www.jstor.org/stable/info/10.2307/40166677

122. Avery ML. Rusty Blackbird (Euphagus carolinus). Poole A, Gill F, editors. Birds North Am Online [Internet] 2013; Available from: http://bna.birds.cornell.edu/BNA/account/Rusty_Blackbird/RECOMMENDED_CITATION.html

123. Williams L. Breeding Behavior of the Brewer Blackbird. Condor [Internet] 1952;54(1):3–47. Available from: http://www.jstor.org/stable/1364526?seq=1#page_scan_tab_contents

124. Martin SG. Brewer's Blackbird (Euphagus cyanocephalus). Poole A, Gill F, editors. Birds North Am Online [Internet] 2002; Available from: http://bna.birds.cornell.edu/BNA/account/Brewers_Blackbird/RECOMMENDED_CITATION.html

125. Granholm S, Mewaldt L, Duke R. BREWER'S BLACKBIRD Euphagus cyanocephalus. In: Zeiner DC, Jr. WFL, Mayer KE, White M, editors. Calif Wildl Habitat Relationships Vol I-III. Sacramento, California: California Depart. of Fish and Game; 1990.

126. Rising JD, Flood. NJ. Baltimore Oriole (Icterus galbula). Poole A, Gill F, editors. Birds North Am Online [Internet] 1998; Available from: http://bna.birds.cornell.edu/BNA/account/Baltimore_Oriole/RECOMMENDED_CITATION.html

127. Scharf WC, Kren J. Orchard Oriole (Icterus spurius). Poole A, Gill F, editors. Birds North Am Online [Internet] 2010; Available from: http://bna.birds.cornell.edu/BNA/account/Orchard_Oriole/RECOMMENDED_CITATION.html

128. Rothstein SI, Verner J, Steven E. Radio-Tracking Confirms a Unique Diurnal Pattern of Spatial Occurrence in the Parasitic Brown-Headed Cowbird. Ecology [Internet] 1984 [cited 2015 Nov 20];65(1):77–88. Available from: http://www.jstor.org/stable/1939460

129. Lowther PE. Brown-headed Cowbird (Molothrus ater). Poole A, Gill F, editors. Birds North Am Online [Internet] 1993; Available from: http://bna.birds.cornell.edu/BNA/account/Brown-headed_Cowbird/RECOMMENDED_CITATION.html

130. Bray OE, Royall WCJ, Guarino JL, Johnson RE. Activities of radio-equipped Common Grackles during fall migration. Wilson Bull [Internet] 1979;91(1):78–87. Available from: https://sora.unm.edu/sites/default/files/journals/wilson/v091n01/p0078-p0087.pdf

131. Peer BD, Bollinger EK. Common Grackle (Quiscalus quiscula). Poole A, Gill F, editors. Birds North Am Online [Internet] 1997; Available from: http://bna.birds.cornell.edu/BNA/account/Common_Grackle/RECOMMENDED_CITATION.html

132. Davis SK, Lanyon WE. Western Meadowlark (Sturnella neglecta). Poole A, Gill F, editors. Birds North Am Online [Internet] 2008; Available from: http://bna.birds.cornell.edu/BNA/account/Western_Meadowlark/RECOMMENDED_CITATION.html

133. Twedt DJ, Crawford. RD. Yellow-headed Blackbird (Xanthocephalus xanthocephalus). Poole A, Gill F, editors. Birds North Am Online [Internet] 1995; Available from: http://bna.birds.cornell.edu/BNA/account/Yellow-headed_Blackbird/RECOMMENDED_CITATION.html

134. Granholm S, Mewaldt L, Duke R. YELLOW-HEADED BLACKBIRD Xanthocephalus xanthocephalus. In: Zeiner DC, Jr. WFL, Mayer KE, White M,

editors. Calif Wildl Habitat Relationships Vol I-III. Sacramento, California: California Depart. of Fish and Game; 1990.

135. Granholm S, Mewaldt L, Duke R. LOGGERHEAD SHRIKE Lanius ludovicianus. In: Zeiner DC, Jr. WFL, Mayer KE, White M, editors. Calif Wildl Habitat Relationships Vol I-III. Sacramento, California: California Depart. of Fish and Game; 1990.

136. Yosef R. Loggerhead Shrike (Lanius ludovicianus). Poole A, Gill F, editors. Birds North Am Online [Internet] 1996; Available from: http://bna.birds.cornell.edu/BNA/account/Loggerhead_Shrike/RECOMMENDED_CITATION.html

137. Smith RJ, Hatch MI, Cimprich DA, Moore FR. Gray Catbird (Dumetella carolinensis). Poole A, Gill F, editors. Birds North Am Online [Internet] 2011; Available from: http://bna.birds.cornell.edu/BNA/account/Gray_Catbird/RECOMMENDED_CITATION.html

138. Farnsworth G, Londono GA, Martin JU, DERRICKSON KC, BREITWISCH R. Northern Mockingbird (Mimus polyglottos). Poole A, Stettenheim P, Gill F, editors. Birds North Am Online [Internet] 2011;(7). Available from: http://bna.birds.cornell.edu/BNA/account/Northern_Mockingbird/RECOMMENDED_CITATION.html

139. Dobkin D, Mewaldt L, Duke R. NORTHERN MOCKINGBIRD Mimus polyglottos. In: Zeiner DC, Jr. WFL, Mayer KE, White M, editors. Calif Wildl Habitat Relationships Vol I-III. Sacramento, California: California Depart. of Fish and Game; 1990.

140. Cavitt JF, Haas CA. Brown Thrasher (Toxostoma rufum). Poole A, Gill F, editors. Birds North Am Online [Internet] 2014; Available from: http://bna.birds.cornell.edu/BNA/account/Brown_Thrasher/RECOMMENDED_CITATION.html

141. Eastman J. The Eastman Guide to Birds: Natural History Accounts for 150 North American Species [Internet]. Stackpole Books; 2000 [cited 2015 Nov 30]. Available from: https://books.google.com/books?id=4s039PKpc4kC&pgis=1 ISBN:081174552X

142. Robbins MB. Display Behavior of Male Sprague's Pipits. Wilson Bull [Internet] 1998 [cited 2015 Nov 12];110(3):435–438. Available from: http://www.jstor.org/stable/4163976

143. Davis SK, Robbins MB, Dale BC. Sprague's Pipit (Anthus spragueii). Poole A, Gill F, editors. Birds North Am Online [Internet] 2014; Available from: http://bna.birds.cornell.edu/BNA/account/Spragues_Pipit/RECOMMENDED_CITATION.html

144. Poole AF, Bierregaard RO, Martell MS. Osprey (Pandion haliaetus). Poole A, Gill F, editors. Birds North Am Online [Internet] 2002; Available from: http://bna.birds.cornell.edu/BNA/account/Osprey/RECOMMENDED_CITATION.html

145. Foote JR, Mennill DJ, Ratcliffe LM, Smith SM. Black-capped Chickadee (Poecile atricapillus). Poole A, Stettenheim P, Gill F, editors. Birds North Am Online [Internet] 2010; Available from: http://bna.birds.cornell.edu/BNA/account/Black-capped_Chickadee/RECOMMENDED_CITATION.html

146. Greenewalt CH. The flight of the Black-capped Chickadee and the White-breasted Nuthatch. Auk [Internet] 1955 [cited 2015 Nov 6];72:1–5. Available from: https://sora.unm.edu/sites/default/files/journals/auk/v072n01/p0001-p0005.pdf

147. Odum EP. Annual Cycle of the Black-Capped Chickadee: 3. Auk [Internet] 1942 Oct [cited 2015 Nov 30];59(4):499–531. Available from: http://www.jstor.org/stable/info/10.2307/4079461

148. Hadley A, Desrochers A. Winter Habitat Use by Boreal Chickadee Flocks in a Managed Forest. Wilson J Ornithol [Internet] 2008 [cited 2015 Nov 30];120(1):139–145. Available from: http://theses.ulaval.ca/archimede/fichiers/23847/ch02.html#d0e269

149. Ficken MS, Mclaren MA, Hailman JP. Boreal Chickadee (Poecile hudsonica). Poole A, Gill F, editors. Birds North Am Online [Internet] 1996; Available from: http://bna.birds.cornell.edu/BNA/account/Boreal_Chickadee/RECOMMENDED_CITATION.html

150. Savignac C. COSEWIC assessment and status report on the Canada warbler, Wilsonia canadensis, in Canad [Internet]. Libr Arch Canada Electron Collect. 2008 [cited 2015 Nov 17]. Available from: http://epe.lac-bac.gc.ca/100/200/301/environment_can/cws-scf/cosewic-cosepac/canada_warbler-e/CW69-14-548-2008E.pdf Archived at: http://www.webcitation.org/6lD2aBxNQ

151. Reitsma L, Goodnow M, Hallworth MT, Conway CJ. Canada Warbler (Wilsonia canadensis). Poole A, Gill F, editors. Birds North Am Online [Internet] 2010; Available from: http://bna.birds.cornell.edu/bna/species/421

152. Berthold P, Gwinner E, Sonnenschein E. Avian Migration [Internet]. Springer Science & Business Media; 2013 [cited 2015 Nov 6]. Available from: https://books.google.com/books?id=zDvvCAAAQBAJ&pgis=1 ISBN:3662059576

153. Ammon EM, Gilbert. WM. Wilson's Warbler (Wilsonia pusilla). Poole A, Gill F, editors. Birds North Am Online [Internet] 1999; Available from: http://bna.birds.cornell.edu/BNA/account/Wilsons_Warbler/RECOMMENDED_CITATION.html

154. Green M, Mewaldt L, Duke R, Winkler D. WILSON'S WARBLER Wilsonia pusilla. In: Zeiner DC, Jr. WFL, Mayer KE, White M, editors. Calif Wildl Habitat Relationships Vol I-III. Sacramento, California: California Depart. of Fish and Game; 1990.

155. Cox GW. A Life History of the Mourning Warbler. Wilson Bull [Internet] 1960 [cited 2015 Nov 17];72(1):5–28. Available from: https://sora.unm.edu/node/127797

156. Pitocchelli J. Mourning Warbler (Oporornis philadelphia). Poole A, Gill F, editors. Birds North Am Online [Internet] 1993; Available from: http://bna.birds.cornell.edu/BNA/account/Mourning_Warbler/RECOMMENDED_CITATION.html

157. Green M, Mewaldt L, Duke R, Winkler D. COMMON YELLOWTHROAT Geothlypis trichas. In: Zeiner DC, Jr. WFL, Mayer KE, White M, editors. Calif Wildl Habitat Relationships Vol I-III. Sacramento, California: California Depart. of Fish and Game; 1990.

158. Guzy MJ, Ritchison. G. Common Yellowthroat (Geothlypis trichas). Poole A, Gill F, editors. Birds North Am Online [Internet] 1999; Available from: http://bna.birds.cornell.edu/BNA/account/Common_Yellowthroat/RECOMMENDED_CITATION.html

159. James RD. Habitat management guidelines for birds of Ontario wetlands, including marshes, swamps and fens or bogs of various types [Internet]. Ontario Minist Nat Resour. 1985. Available from: http://wbn.scholarsportal.info/node/16950 ISBN:0-7794-2349-6

160. Kricher JC. Black-and-white Warbler (Mniotilta varia). Poole A, Gill F, editors. Birds North Am Online [Internet] 2014; Available from: http://bna.birds.cornell.edu/BNA/account/Black-and-white_Warbler/RECOMMENDED_CITATION.html

161. Pitocchelli J, Jones J, Jones D, Bouchie J. Connecticut Warbler (Oporornis agilis). Poole A, Gill F, editors. Birds North Am Online [Internet] 2012; Available from: http://bna.birds.cornell.edu/BNA/account/Connecticut_Warbler/RECOMMENDED_CITATION.html

162. Gilbert WM, Sogge MK, Van Riper III C. Orange-crowned Warbler (Vermivora celata). Poole A, Gill F, editors. Birds North Am Online [Internet] 2010; Available from: http://bna.birds.cornell.edu/BNA/account/Orange-crowned_Warbler/RECOMMENDED_CITATION.html

163. Green M, Mewaldt L, Duke R, Winkler D. ORANGE-CROWNED WARBLER Vermivora celata. In: Zeiner DC, Jr. WFL, Mayer KE, White M, editors. Calif Wildl Habitat Relationships Vol I-III. Sacramento, California: California Depart. of Fish and Game; 1990.

164. Adlinger K, Bakermans M, Larkin J, Lehman J, Tisdale A. Monitoring and Evaluating Golden-winged Warbler Use of Breeding Habitat [Internet]. 2014. Available from: http://www.nrcs.usda.gov/Internet/FSE_DOCUMENTS/nrcseprd331009.pdf. Archived at: http://www.webcitation.org/6lCy8jP7r

165. Rimmer CC, Mcfarland. KP. Tennessee Warbler (Vermivora peregrina). Poole A, Gill F, editors. Birds North Am Online [Internet] 2012; Available from: http://bna.birds.cornell.edu/BNA/account/Tennessee_Warbler/RECOMMENDED_CITATION.html

166. Lowther PE, Williams JM. Nashville Warbler (Vermivora ruficapilla). Poole A, Gill F, editors. Birds North Am Online [Internet] 2011; Available from: http://bna.birds.cornell.edu/BNA/account/Nashville_Warbler/RECOMMENDED_CITATION.html

167. Whitaker DM, Eaton SW. Northern Waterthrush (Seiurus noveboracensis). Poole A, Gill F, editors. Birds North Am Online [Internet] 2014; Available from: http://bna.birds.cornell.edu/BNA/account/Northern_Waterthrush/RECOMMENDED_CITATION.html

168. Porneluzi P, Van Horn MA, Donovan TM. Ovenbird (Seiurus aurocapilla). Poole A, Gill F, editors. Birds North Am Online [Internet] 2011;(88). Available from: http://bna.birds.cornell.edu/BNA/species/088/articles/behavior

169. Moldenhauer RR, Regelski DJ. Northern Parula (Parula americana). Poole A, Gill F, editors. Birds North Am Online [Internet] 2012; Available from: http://bna.birds.cornell.edu/BNA/account/Northern_Parula/RECOMMENDED_CITATION.html

170. Venier L, Holmes S, Williams JM. Bay-breasted Warbler (Dendroica castanea). Poole A, Gill F, editors. Birds North Am Online [Internet] 2011; Available from: http://bna.birds.cornell.edu/BNA/account/Bay-breasted_Warbler/RECOMMENDED_CITATION.html

171. Slager DL, Rodewald PG. Disjunct Nocturnal Roosting by a Yellow-rumped Warbler (Setophaga coronata) during Migratory Stopover. Wilson J Ornithol [Internet] 2015 Mar;127(1):109–114. Available from: http://www.bioone.org/doi/10.1676/14-038.1 DOI:10.1676/14-038.1

172. Hunt PD, Flaspohler DJ. Yellow-rumped Warbler (Dendroica coronata). Poole A, Gill F, editors. Birds North Am Online [Internet] 1998; Available from: http://bna.birds.cornell.edu/bna/species/376

173. Morse DH. Blackburnian Warbler (Dendroica fusca). Poole A, editor. Birds North Am Online [Internet] 2004; Available from: http://bna.birds.cornell.edu/BNA/account/Blackburnian_Warbler/RECOMMENDED_CITATION.html

174. Dunn E, Hall GA. Magnolia Warbler (Dendroica magnolia). Poole A, Gill F, editors. Birds North Am Online [Internet] 1994; Available from: http://bna.birds.cornell.edu/BNA/account/Magnolia_Warbler/RECOMMENDED_CITATION.html

175. Wilson J, Herbert. W. Palm Warbler (Dendroica palmarum). Poole A, Gill F, editors. Birds North Am Online [Internet] 1996; Available from: http://bna.birds.cornell.edu/BNA/account/Palm_Warbler/RECOMMENDED_CITATION.html

176. Byers BE, Richardson M, Brauning DW. Chestnut-sided Warbler (Dendroica pensylvanica). Poole A, Gill F, editors. Birds North Am Online [Internet] 2013; Available from: http://bna.birds.cornell.edu/BNA/account/Chestnut-sided_Warbler/RECOMMENDED_CITATION.html

177. Lowther PE, Celada C, Klein NK, Rimmer CC, Spector DA. Yellow Warbler (Dendroica petechia). Poole A, Gill F, editors. Birds North Am Online [Internet] 1999; Available from: http://bna.birds.cornell.edu/BNA/account/Yellow_Warbler/RECOMMENDED_CITATION.html

178. Latta SC., Sondreal ML. Observations on the Abundance, Site Persistence, Home Range, Foraging, and Nesting of the Pine Warbler on Hispaniola, and First Record of Ground Nesting for This Species. Ornitol Neotrop [Internet] 1999 [cited 2015 Nov 18];10(1):43–54. Available from: https://sora.unm.edu/node/119317

179. Rodewald PG, Withgott JH, Smith KG. Pine Warbler (Dendroica pinus). Poole A, Gill F, editors. Birds North Am Online [Internet] 1999; Available from: http://bna.birds.cornell.edu/BNA/account/Pine_Warbler/RECOMMENDED_CITATION.html

180. Sherry TW, Holmes RT. American Redstart (Setophaga ruticilla). Poole A, Gill F, editors. Birds North Am Online [Internet] 1997;(277). Available from: http://bna.birds.cornell.edu/BNA/species/277/articles/behavior

181. Morse B. Weather and Migrating Fall Warblers in Eastern Washington. Washingt Ornithol Soc News [Internet] 2001 [cited 2015 Nov 17];(74). Available from: http://www.wos.org/issue74.pdf

182. DeLuca W, Holberton R, Hunt PD, Eliason BC. Blackpoll Warbler (Dendroica striata). Poole A, Gill F, editors. Birds North Am Online [Internet] 2013; Available from: http://bna.birds.cornell.edu/BNA/account/Blackpoll_Warbler/RECOMMENDED_CITATION.html

183. Baltz ME, Latta SC. Cape May Warbler (Dendroica tigrina). Poole A, Gill F, editors. Birds North Am Online [Internet] 1998; Available from: http://bna.birds.cornell.edu/BNA/account/Cape_May_Warbler/RECOMMENDED_CITATION.html

184. Morse DH, Poole AF. Black-throated Green Warbler (Dendroica virens). Poole A, Gill F, editors. Birds North Am Online [Internet] 1993; Available from: http://bna.birds.cornell.edu/BNA/account/Black-throated_Green_Warbler/RECOMMENDED_CITATION.html

185. Confer JL, Hartman P, Roth A. Golden-winged Warbler (Vermivora chrysoptera). Poole A, Stettenheim P, Gill F, editors. Birds North Am Online [Internet] 2011; Available from: http://bna.birds.cornell.edu/BNA/account/Golden-winged_Warbler/RECOMMENDED_CITATION.html

186. Lowther PE, Cink. CL. House Sparrow (Passer domesticus). Poole A, Stettenheim P, Gill F, editors. Birds North Am Online [Internet] 2006; Available from: http://bna.birds.cornell.edu/BNA/account/House_Sparrow/RECOMMENDED_CITATION.html

187. Granholm S, Mewaldt L, Duke R. HOUSE SPARROW Passer domesticus. In: Zeiner DC, Jr. WFL, Mayer KE, White M, editors. Calif Wildl Habitat Relationships Vol I-III. Sacramento, California: California Depart. of Fish and Game; 1990.

188. Ahlborn G, Johnson N, Ahlborn G. RUFFED GROUSE Bonasa umbellus. In: Zeiner DC, Jr. WFL, Mayer KE, White M, editors. Calif Wildl Habitat Relationships Vol I-III. Sacramento, California: California Depart. of Fish and Game; 1990.

189. Rusch DH, Destefano S, Reynolds MC, Lauten D. Ruffed Grouse (Bonasa umbellus). Poole A, Gill F, editors. Birds North Am Online [Internet] 2000; Available from: http://bna.birds.cornell.edu/BNA/account/Ruffed_Grouse/RECOMMENDED_CITATION.html

190. McRoberts JT, Wallace MC, Eaton SW. Wild Turkey (Meleagris gallopavo). Poole A, Stettenheim P, Gill F, editors. Birds North Am Online [Internet] 2014; Available from: http://bna.birds.cornell.edu/BNA/account/Wild_Turkey/RECOMMENDED_CITATION.html

191. Novoa C, Dumas S, Resseguier J. Home-range size of Pyrenean grey partridges Perdix perdix hispaniensis during the breeding season. Wildlife Biol. 2006. p. 11–18.

192. Iowa Department of Natural Resources. Gray (Hungarian) partridge (Perdix perdix) [Internet]. 2001 [cited 2015 Nov 6]. Available from: http://www.iowadnr.gov/Portals/idnr/uploads/education/Species/birds/Hun.pdf. Archived at: http://www.webcitation.org/6lCyR61f6

193. Carroll JP. Gray Partridge (Perdix perdix). Poole A, Gill F, editors. Birds North Am Online [Internet] 1993; Available from: http://bna.birds.cornell.edu/BNA/account/Gray_Partridge/RECOMMENDED_CITATION.html

194. New World Encyclopedia contributors. Pheasant [Internet]. New World Encycl. 2009 [cited 2015 Nov 26]. Available from: http://www.newworldencyclopedia.org/entry/Pheasant Archived at: http://www.webcitation.org/6lD38ZdUX

195. Ahlborn G, Johnson N, Ahlborn G. RING-NECKED PHEASANT Phasianus colchicus. In: Zeiner DC, Jr. WFL, Mayer KE, White M, editors. Calif Wildl Habitat Relationships Vol I-III. Sacramento, California: California Depart. of Fish and Game; 1990.

196. Giudice JH, Ratti JT. Ring-necked Pheasant (Phasianus colchicus). Poole A, Gill F, editors. Birds North Am Online [Internet] 2001; Available from: http://bna.birds.cornell.edu/BNA/account/Ring-necked_Pheasant/RECOMMENDED_CITATION.html

197. Connelly JW, Gratson MW, Reese KP. Sharp-tailed Grouse (Tympanuchus phasianellus). Poole A, Gill F, editors. Birds North Am Online [Internet] 1998; Available from: http://bna.birds.cornell.edu/BNA/account/Sharp-tailed_Grouse/RECOMMENDED_CITATION.html

198. Wiebe KL, Moore WS. Northern Flicker (Colaptes auratus). Poole A, Gill F, editors. Birds North Am Online [Internet] 2008; Available from: http://bna.birds.cornell.edu/BNA/account/Northern_Flicker/RECOMMENDED_CITATION.html

199. Tobalske BW. Scaling of Muscle Composition, Wing Morphology, and Intermittent Flight Behavior in Woodpeckers. Auk [Internet] 1996 Jan [cited 2015 Nov 5];113(1):151–177. Available from: http://www.jstor.org/stable/info/10.2307/4088943 DOI:10.2307/4088943

200. Bull EL, Jackson JA. Pileated Woodpecker (Dryocopus pileatus). Poole A, Gill F, editors. Birds North Am Online [Internet] 1995; Available from: http://bna.birds.cornell.edu/BNA/account/Pileated_Woodpecker/RECOMMENDED_CITATION.html

201. Vierling KT, Saab VA, Tobalske BW. Lewis's Woodpecker (Melanerpes lewis). Poole A, Gill F, editors. Birds North Am Online [Internet] 2013; Available from: http://bna.birds.cornell.edu/BNA/account/Lewiss_Woodpecker/RECOMMENDED_CITATION.html

202. Frei B, Smith KG, Withgott JH, Rodewald PG. Red-headed Woodpecker (Melanerpes erythrocephalus). Poole A, Gill F, editors. Birds North Am Online [Internet] 2015; Available from: http://bna.birds.cornell.edu/BNA/account/Red-headed_Woodpecker/RECOMMENDED_CITATION.html

203. Leonard, Jr. DL. American Three-toed Woodpecker (Picoides dorsalis). Poole A, Gill F, editors. Birds North Am Online [Internet] 2001; Available from: http://bna.birds.cornell.edu/BNA/account/American_Three-toed_Woodpecker/RECOMMENDED_CITATION.html

204. Jackson JA, Ouellet. HR. Downy Woodpecker (Picoides pubescens). Poole A, Gill F, editors. Birds North Am Online [Internet] 2002; Available from: http://bna.birds.cornell.edu/BNA/account/Downy_Woodpecker/RECOMMENDED_CITATION.html

205. Kilham L. Courtship and Territorial Behaviour of Hairy Woopeckers. Auk [Internet] 1960;77(3):259–270. Available from: http://www.jstor.org/stable/pdf/4082482

206. Covert-Bratland KA, Block WM, Theimer TC. Hairy woodpecker winter ecology in ponderosa pine forests representing different ages since wildfire. J Wildl Manage [Internet] 2006 [cited 2015 Nov 6];70(5):1379–1392. Available from: http://www.treesearch.fs.fed.us/pubs/26328

207. Jackson JA, Ouellet HR, Jackson BJ. Hairy Woodpecker (Picoides villosus). Poole A, Gill F, editors. Birds North Am Online [Internet] 2002; Available from: http://bna.birds.cornell.edu/BNA/account/Hairy_Woodpecker/RECOMMENDED_CITATION.html

208. Kilham L. Reproductive Behavior of Hairy Woodpeckers I. Pair Formation and Courtship. Wilson Bull [Internet] 1966;78(3):251–265. Available from: http://www.jstor.org/stable/4159500

209. Swanson DL, Ingold JL, Wallace GE. Ruby-crowned Kinglet (Regulus calendula). Poole A, Gill F, editors. Birds North Am Online [Internet] 1994;

Available from: http://bna.birds.cornell.edu/BNA/account/Ruby-crowned_Kinglet/RECOMMENDED_CITATION.html

210. Swanson DL, Ingold JL, Galati R. Golden-crowned Kinglet (Regulus satrapa). Poole A, Gill F, editors. Birds North Am Online [Internet] 2012; Available from: http://bna.birds.cornell.edu/BNA/account/Golden-crowned_Kinglet/RECOMMENDED_CITATION.html

211. Ghalambor CK, Martin TE. Red-breasted Nuthatch (Sitta canadensis). Poole A, Gill F, editors. Birds North Am Online [Internet] 1999; Available from: http://bna.birds.cornell.edu/BNA/account/Red-breasted_Nuthatch/RECOMMENDED_CITATION.html

212. Ball SC. Migration of Red-Breasted Nuthatches in Gaspe. Ecol Monogr [Internet] 1947 Feb;17(4):501–533. Available from: http://doi.wiley.com/10.2307/1948598 DOI:10.2307/1948598

213. Grubb, Jr. TC, Pravosudov V V. White-breasted Nuthatch (Sitta carolinensis). Poole A, Gill F, editors. Birds North Am Online [Internet] 2008; Available from: http://bna.birds.cornell.edu/BNA/account/White-breasted_Nuthatch/RECOMMENDED_CITATION.html

214. Simply Wild Canada. Owls of Canada [Internet]. [cited 2015 Nov 5]. Available from: http://www.simplywildcanada.com/wild-species/birds-of-canada/owls-of-canada/ Archived at: http://www.webcitation.org/6lD3RVJm6

215. Wiggins DA, Holt DW, Leasure SM. Short-eared Owl (Asio flammeus). Poole A, Gill F, editors. Birds North Am Online [Internet] 2006; Available from: http://bna.birds.cornell.edu/BNA/account/Short-eared_Owl/RECOMMENDED_CITATION.html

216. Bennett JR, Bloom PH. Range and Habitat Use by Great Horned Owls (Bubo virginianus) in Southern California. J Raptor Res [Internet] 2005;39(2):119–126. Available from: https://pubs.er.usgs.gov/publication/70029641

217. Artuso C, Houston CS, Smith DG, Rohner C. Great Horned Owl (Bubo virginianus). Poole A, Gill F, editors. Birds North Am Online [Internet] 2014; Available from: http://bna.birds.cornell.edu/BNA/account/Great_Horned_Owl/RECOMMENDED_CITATION.html

218. Makarieva AM, Gorshkov VG, Li BL. Why do population density and inverse home range scale differently with body size?: Implications for ecosystem stability. Ecol Complex 2005;2(3):259–271.

219. Mazur KM, James PC. Barred Owl (Strix varia). Poole A, Gill F, editors. Birds North Am Online [Internet] 2000; Available from: http://bna.birds.cornell.edu/BNA/account/Barred_Owl/RECOMMENDED_CITATION.html

220. Kroeger RA, Grushka HD, Helvey TC. Low Speed Aerodynamics for Ultra-Quiet Flight [Internet]. 1972 Mar. Available from: http://oai.dtic.mil/oai/oai?verb=getRecord&metadataPrefix=html&identifier=AD0893426 Archived at: http://www.webcitation.org/6lD3lBJMn

221. Cabe PR. European Starling (Sturnus vulgaris). Poole A, Gill F, editors. Birds North Am Online [Internet] 1993; Available from: http://bna.birds.cornell.edu/BNA/account/European_Starling/RECOMMENDED_CITATION.html

222. Granholm S, Mewaldt L, Duke R. EUROPEAN STARLING Sturnus vulgaris. In: Zeiner DC, Jr. WFL, Mayer KE, White M, editors. Calif Wildl Habitat Relationships Vol I-III. Sacramento, California: California Depart. of Fish and Game; 1990.

223. Weidensaul S, Robinson TR, Sargent RR, Sargent MB. Ruby-throated Hummingbird (Archilochus colubris). Poole A, Gill F, editors. Birds North Am Online [Internet] 2013; Available from: http://bna.birds.cornell.edu/BNA/account/Ruby-throated_Hummingbird/RECOMMENDED_CITATION.html

224. Chambers L. Ruby-throated Hummingbird [Internet]. [cited 2015 Nov 13]. Available from: http://www.hummingbirds.net/rubythroated.html. Archived at: http://www.webcitation.org/6lD3wmHf8

225. Rousseu F, Charette Y, Bélisle M. Resource defense and monopolization in a marked population of ruby-throated hummingbirds (Archilochus colubris). Ecol Evol John Wiley and Sons Ltd; 2014;4(6):776–793.

226. Texas Parks and Wildlife Department. Urban Wildlife Fact Sheet Set [Internet]. [cited 2015 Nov 13]. Available from: https://tpwd.texas.gov/publications/pwdpubs/media/pwd_lf_k0700_0167.pdf Archived at: http://www.webcitation.org/6lCxcCrDf

227. Kroodsma DE, Verner J. Marsh Wren (Cistothorus palustris). Poole A, Gill F, editors. Birds North Am Online [Internet] 2014; Available from: http://bna.birds.cornell.edu/BNA/account/Marsh_Wren/RECOMMENDED_CITATION.html

228. Gutzwiller KJ, Anderson SH. Habitat Suitability Index Models: Marsh Wren. Biol Rep [Internet] 1987;82(10.139):13. Available from: http://www.nwrc.usgs.gov/wdb/pub/hsi/hsi-139.pdf

229. Hartman CA, Ackerman JT, Herring G, Isanhart J, Herzog M. Marsh wrens as bioindicators of mercury in wetlands of Great Salt Lake: do blood and feathers reflect site-specific exposure risk to bird reproduction? Environ Sci Technol [Internet] American Chemical Society; 2013 Jun 18 [cited 2015 Nov 18];47(12):6597–605. Available from: http://dx.doi.org/10.1021/es400910x PMID: 23692510

230. Burns JT. Nests, Territories, and Reproduction of Sedge Wrens (Cistothorus platensis). Wilson Bull [Internet] 1982 [cited 2015 Nov 18];94(3):338–349. Available from: http://www.jstor.org/stable/4161644

231. Herkert JR, Kroodsma DE, Gibbs JP. Sedge Wren (Cistothorus platensis). Poole A, Gill F, editors. Birds North Am Online [Internet] 2001; Available from: http://bna.birds.cornell.edu/BNA/account/Sedge_Wren/RECOMMENDED_CITATION.html

232. Fujikawa A. Home range of Coryphaspiza melanotis and Cistothorus platensis in the central Brasil and a review of home ranges and territories of birds in the Neotropics [Internet]. Universidade de São Paulo; 2012 [cited 2015 Nov 18]. Available from: http://www.teses.usp.br/teses/disponiveis/41/41134/tde-17012012-133318/

233. Kroodsma DE. Coexistence of Bewick's Wrens and House Wrens in Oregon. Auk [Internet] 1973 [cited 2015 Dec 1];90(2):341–352. Available from: http://www.jstor.org/stable/4084301

234. Johnson LS. House Wren (Troglodytes aedon). Poole A, Gill F, editors. Birds North Am Online [Internet] 2014; Available from: http://bna.birds.cornell.edu/bna/species/380

235. Hejl SJ, Holmes JA, Kroodsma DE. Winter Wren (Troglodytes troglodytes). Poole A, Gill F, editors. Birds North Am Online [Internet] 2002; Available from: http://bna.birds.cornell.edu/BNA/account/Winter_Wren/RECOMMENDED_CITATION.html

236. Bevier LR, Poole AF, Moskoff W. Veery (Catharus fuscescens). Poole A, editor. Birds North Am Online [Internet] 2005; Available from: http://bna.birds.cornell.edu/BNA/account/Veery/RECOMMENDED_CITATION.html

237. Bayly NJ, Gómez C, Hobson KA. Energy reserves stored by migrating Gray-cheeked Thrushes Catharus minimus at a spring stopover site in northern Colombia are sufficient for a long-distance flight to North America. Ishtiaq F, editor. Ibis (Lond 1859) [Internet] 2013 Apr 16 [cited 2015 Nov 18];155(2):271–283. Available from: http://doi.wiley.com/10.1111/ibi.12029

238. Dellinger R, Wood PB, Jones PW, Donovan TM. Hermit Thrush (Catharus guttatus). Poole A, Gill F, editors. Birds North Am Online [Internet] 2012; Available from: http://bna.birds.cornell.edu/BNA/account/Hermit_Thrush/RECOMMENDED_CITATION.html

239. Rinaldi T, Worland M. Conservation Assessment for Swainson’s Thrush (Catharus ustulatus) [Internet]. USDA For Serv East Reg. 2004 [cited 2015 Nov 18]. Available from: http://www.fs.usda.gov/Internet/FSE_DOCUMENTS/fsm91_054321.pdf. Archived at: http://www.webcitation.org/6lCyjP0XW

240. Mack DE, Yong W. Swainson’s Thrush (Catharus ustulatus). Poole A, Gill F, editors. Birds North Am Online [Internet] 2000; Available from: http://bna.birds.cornell.edu/BNA/account/Swainsons_Thrush/RECOMMENDED_CITATION.html

241. Power HW, Lombard MP. Mountain Bluebird (Sialia currucoides). Poole A, Gill F, editors. Birds North Am Online [Internet] 1996; Available from: http://bna.birds.cornell.edu/BNA/account/Mountain_Bluebird/RECOMMENDED_CITATION.html

242. Gaines D, Mewaldt L, Duke R. MOUNTAIN BLUEBIRD Sialia currucoides. In: Zeiner DC, Jr. WFL, Mayer KE, White M, editors. Calif Wildl Habitat Relationships Vol I-III. Sacramento, California: California Depart. of Fish and Game; 1990.

243. Gowaty PA, Plissner JH. Eastern Bluebird (Sialia sialis). Poole A, Gill F, editors. Birds North Am Online [Internet] 2015;(381). Available from: http://bna.birds.cornell.edu/bna/species/381/articles/demography

244. Hirth DH, Hester AE, Greeley F. Dispersal and Flocking of Marked Young Robins (Turdus M. Migratorius) after Fledging. Bird-Banding [Internet] 1969 [cited 2015 Dec 1];40(3):208. Available from: http://www.jstor.org/stable/10.2307/4511580?origin=crossref

245. Vanderhoff N, Sallabanks R, James FC. American Robin (Turdus migratorius). Poole A, Gill F, editors. Birds North Am Online [Internet] 2014; Available from: http://bna.birds.cornell.edu/BNA/account/American_Robin/RECOMMENDED_CITATION.html

246. Diuk-Wasser MA, Molaei G, Simpson JE, Folsom-O’Keefe CM, Armstrong PM, Andreadis TG. Avian communal roosts as amplification foci for West Nile virus in urban areas in Northeastern United States. Am J Trop Med Hyg 2010;82(2):337–343.

247. Granholm S, Mewaldt L, Duke R. AMERICAN ROBIN Turdus migratorius. In: Zeiner DC, Jr. WFL, Mayer KE, White M, editors. Calif Wildl Habitat Relationships Vol I-III. Sacramento, California: California Depart. of Fish and Game; 1990.

248. Muijres FT, Bowlin MS, Johansson LC, Hedenström A. Vortex wake, downwash distribution, aerodynamic performance and wingbeat kinematics in slow-flying pied flycatchers. J R Soc Interface [Internet] 2012 Feb 7 [cited 2015 Nov 6];9(67):292–303. Available from: https://www.ncbi.nlm.nih.gov/pmc/articles/PMC3243385/ PMID: 21676971

249. Altman B, Sallabanks R. Olive-sided Flycatcher (Contopus cooperi). Poole A, Gill F, editors. Birds North Am Online [Internet] 2012; Available from: http://bna.birds.cornell.edu/BNA/account/Olive-sided_Flycatcher/RECOMMENDED_CITATION.html

250. Otto P. What’s a stranded wood-pewee to do? [Internet]. Kane Cty Chron. 2012 [cited 2015 Nov 18]. Available from: http://www.kcchronicle.com/mobile/article.xml/articles/2012/11/02/r_6zeodgkeslmsrmtheo2qvg/index.xml Archived at: http://www.webcitation.org/6lD4PICRO

251. Bemis C, Rising JD. Western Wood-Pewee (Contopus sordidulus). Poole A, Gill F, editors. Birds North Am Online [Internet] 1999; Available from: http://bna.birds.cornell.edu/BNA/account/Western_Wood-Pewee/RECOMMENDED_CITATION.html

252. Eckhardt RC. The Adaptive Syndromes of Two Guilds of Insectivorous Birds in the Colorado Rocky Mountains. Ecol Monogr [Internet] 1979 [cited 2015 Nov 18];49(2):129–149. Available from: http://www.jstor.org/stable/1942510

253. McCarty JP. Eastern Wood-Pewee (Contopus virens). Poole A, Gill F, editors. Birds North Am Online [Internet] 1996; Available from: http://bna.birds.cornell.edu/BNA/account/Eastern_Wood-Pewee/RECOMMENDED_CITATION.html

254. Lowther PE. Alder Flycatcher (Empidonax alnorum). Poole A, Gill F, editors. Birds North Am Online [Internet] 1999; Available from: http://bna.birds.cornell.edu/BNA/account/Alder_Flycatcher/RECOMMENDED_CITATION.html

255. Dunson B. The Incredible Flight of a Willow Flycatcher [Internet]. Galax Gaz. 2014 [cited 2015 Nov 18]. Available from: http://www.galaxgazette.com/content/incredible-flight-willow-flycatcher Archived at: http://www.webcitation.org/6lD4ZehQb

256. Gross DA, Lowther PE. Yellow-bellied Flycatcher (Empidonax flaviventris). Poole A, Gill F, editors. Birds North Am Online [Internet] 2011; Available from: http://bna.birds.cornell.edu/BNA/account/Yellow-bellied_Flycatcher/RECOMMENDED_CITATION.html

257. Doyon F, Higgelke PE, MacLeod HL. Least Flycatcher (Empidonax minimus) [Internet]. Prep Millar West For Prod Biodivers Assess Proj - ISFORT. 2000 [cited 2015 Nov 18]. Available from: http://isfort.uqo.ca/sites/isfort.uqo.ca/files/fichiers/publications_ISFORT/least_flycatcher_hsm.pdf. Archived

at: http://www.webcitation.org/6lD4d2d7K

258. Tarof S, Briskie J V. Least Flycatcher (Empidonax minimus). Poole A, Gill F, editors. Birds North Am Online [Internet] 2008; Available from: http://bna.birds.cornell.edu/BNA/account/Least_Flycatcher/RECOMMENDED_CITATION.html

259. Cardinal SN, Paxton EH. Home Range, movement, and habitat use of the Southwestern Willow Flycatcher, Roosevelt Lake, AZ - 2004 [Internet]. US Geol Surv Rep to US Bur Reclamation, Phoenix. 2005 [cited 2015 Nov 18]. p. 26. Available from: http://sbsc.wr.usgs.gov/cprs/research/projects/swwf/Reports/telemetry2004report.pdf

260. Sedgwick JA. Willow Flycatcher (Empidonax traillii). Poole A, Gill F, editors. Birds North Am Online [Internet] 2000; Available from: http://bna.birds.cornell.edu/BNA/account/Willow_Flycatcher/RECOMMENDED_CITATION.html

261. Miller KE, Lanyon WE. Great Crested Flycatcher (Myiarchus crinitus). Poole A, Gill F, editors. Birds North Am Online [Internet] 1997; Available from: http://bna.birds.cornell.edu/BNA/account/Great_Crested_Flycatcher/RECOMMENDED_CITATION.html

262. Weeks Jr. HP. Eastern Phoebe (Sayornis phoebe). Poole A, Gill F, editors. Birds North Am Online [Internet] 2011; Available from: http://bna.birds.cornell.edu/BNA/account/Eastern_Phoebe/RECOMMENDED_CITATION.html

263. Johnston DW. Niche Relationships among Some Deciduous Forest Flycatchers. Auk [Internet] 1971;88(4):796–804. Available from: http://www.jstor.org/stable/4083838

264. Gaines D, Mewaldt L, Duke R. SAY'S PHOEBE Sayornis saya. In: Zeiner DC, Jr. WFL, Mayer KE, White M, editors. Calif Wildl Habitat Relationships Vol I-III. Sacramento, California: California Depart. of Fish and Game; 1990.

265. Schukman JM, Wolf BO. Say's Phoebe (Sayornis saya). Poole A, Gill F, editors. Birds North Am Online [Internet] 1998; Available from: http://bna.birds.cornell.edu/bna/species/374

266. Murphy MT. Eastern Kingbird (Tyrannus tyrannus). Poole A, Gill F, editors. Birds North Am Online [Internet] 1996; Available from: http://bna.birds.cornell.edu/BNA/account/Eastern_Kingbird/RECOMMENDED_CITATION.html

267. Gamble LR, Bergin TM. Western Kingbird (Tyrannus verticalis). Poole A, Gill F, editors. Birds North Am Online [Internet] 2012; Available from: http://bna.birds.cornell.edu/BNA/account/Western_Kingbird/RECOMMENDED_CITATION.html

268. Hespenheide HA. Competition and the Genus Tyrannus. Wilson Bull [Internet] 1964 [cited 2015 Dec 1];76(3):265–281. Available from: https://sora.unm.edu/node/128154

269. Rodewald PG, James. RD. Yellow-throated Vireo (Vireo flavifrons). Poole A, Gill F, editors. Birds North Am Online [Internet] 2011; Available from: http://bna.birds.cornell.edu/BNA/account/Yellow-throated_Vireo/RECOMMENDED_CITATION.html

270. James RD. Foraging Behavior and Habitat Selection of Three Species of Vireos in Southern Ontario. Wilson Bull [Internet] 1976 [cited 2015 Dec 1];88(1):62–75. Available from: https://sora.unm.edu/node/129254

271. Sterling R. Vireo gilvus: eastern warbling-vireo [Internet]. Anim Divers Web. 2011 [cited 2015 Dec 1]. Available from: http://www.biokids.umich.edu/critters/Vireo_gilvus/ Archived at: http://www.webcitation.org/6lD4knNii

272. Gardali T, Ballard G. Warbling Vireo (Vireo gilvus). Poole A, Gill F, editors. Birds North Am Online [Internet] 2000; Available from: http://bna.birds.cornell.edu/BNA/account/Warbling_Vireo/RECOMMENDED_CITATION.html

273. Cimprich DA, Moore FR, Guilfoyle MP. Red-eyed Vireo (Vireo olivaceus). Poole A, Gill F, editors. Birds North Am Online [Internet] 2000; Available from: http://bna.birds.cornell.edu/BNA/account/Red-eyed_Vireo/RECOMMENDED_CITATION.html

274. Moskoff W, Robinson SK. Philadelphia Vireo (Vireo philadelphicus). Poole A, Gill F, editors. Birds North Am Online [Internet] 2011; Available from: http://bna.birds.cornell.edu/BNA/account/Philadelphia_Vireo/RECOMMENDED_CITATION.html

275. Morton E, James RD. Blue-headed Vireo (Vireo solitarius). Poole A, Gill F, editors. Birds North Am Online [Internet] 2014; Available from: http://bna.birds.cornell.edu/bna/species/379
